# Precise Synthesis of Ester-Functionalized Cyclo[6]- and Cyclo[7]furans

**DOI:** 10.1021/acs.joc.5c00526

**Published:** 2025-07-07

**Authors:** Dhruv Sharma, Leticia Maria Pequeno Madureira, Tomasz Kowalewski, Kevin J. T. Noonan

**Affiliations:** Department of Chemistry, 6612 Carnegie Mellon University, 4400 Fifth Ave., Pittsburgh, Pennsylvania 15213, United States

## Abstract

Shape-persistent conjugated macrocycles have attracted interest for their unique optoelectronic and self-assembly properties, but the syntheses to obtain these structures can be laborious. In this work, we describe the straightforward synthesis of a recently discovered class of macrocycle, the cyclo­[*n*]­furan, using Suzuki–Miyaura cross-coupling of a simple aromatic monomer. We demonstrate that the combination of hexyl 2-bromo-5-(boronic acid pinacol ester)­furan-3-carboxylate with tris­(dibenzylidene­acetone)­dipalladium(0), tri-*tert*-butylphosphonium tetrafluoroborate and cesium fluoride leads to cyclo[6]- and cyclo[7]­furan esters in 45% yield (28% and 17%, respectively). Crude ^1^H NMR spectroscopy revealed that total conversion to macrocycles was 52 ± 6% over 3 runs, highlighting the robustness of this protocol for cyclofuran synthesis. The oligomerizations are rapid, and model compound studies suggest that a chain-growth mechanism may be operative. The hexyl-substituted cyclo­[*n*]­furan esters (*n* = 6 and 7) are separable via column chromatography. The unique optical and electronic features for each cycle can be partially explained by the size difference for the two systems, as well as the increased conformational flexibility for the larger, ester-functionalized cyclo[7]­furan.

## Graphical abstract

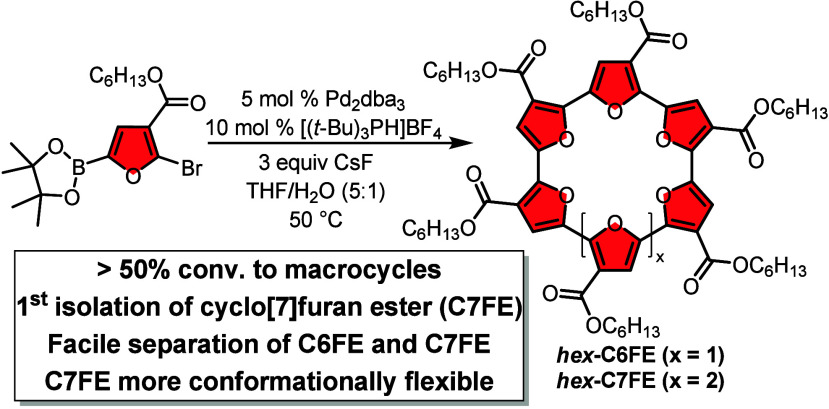

## Introduction

Conjugated cyclic macromolecules comprised only of aromatic rings, have emerged as a novel class of organic electronic material.^1–4^ Benzene, pyrrole and thiophene have been the most common building blocks to construct macrocyclic derivatives, particularly with no spacers. This includes the *meta* ^5^ and *para* ^6^ [*n*]­cyclophenylenes ([*n*]­CMPs or [*n*]­CPPs), the cyclo­[*n*]­pyrroles (CnPs)^7,8^ and the cyclo­[*n*]­thiophenes (CnTs).^9–11^ The shape, structure and properties of the macrocycles are dependent on the building block, the number of repeat units, and the substitution pattern of the repeat unit (Figure 1), which leads to questions regarding aromaticity, optical/redox properties, and solid-state organization.^12–18^

**Figure 1:**
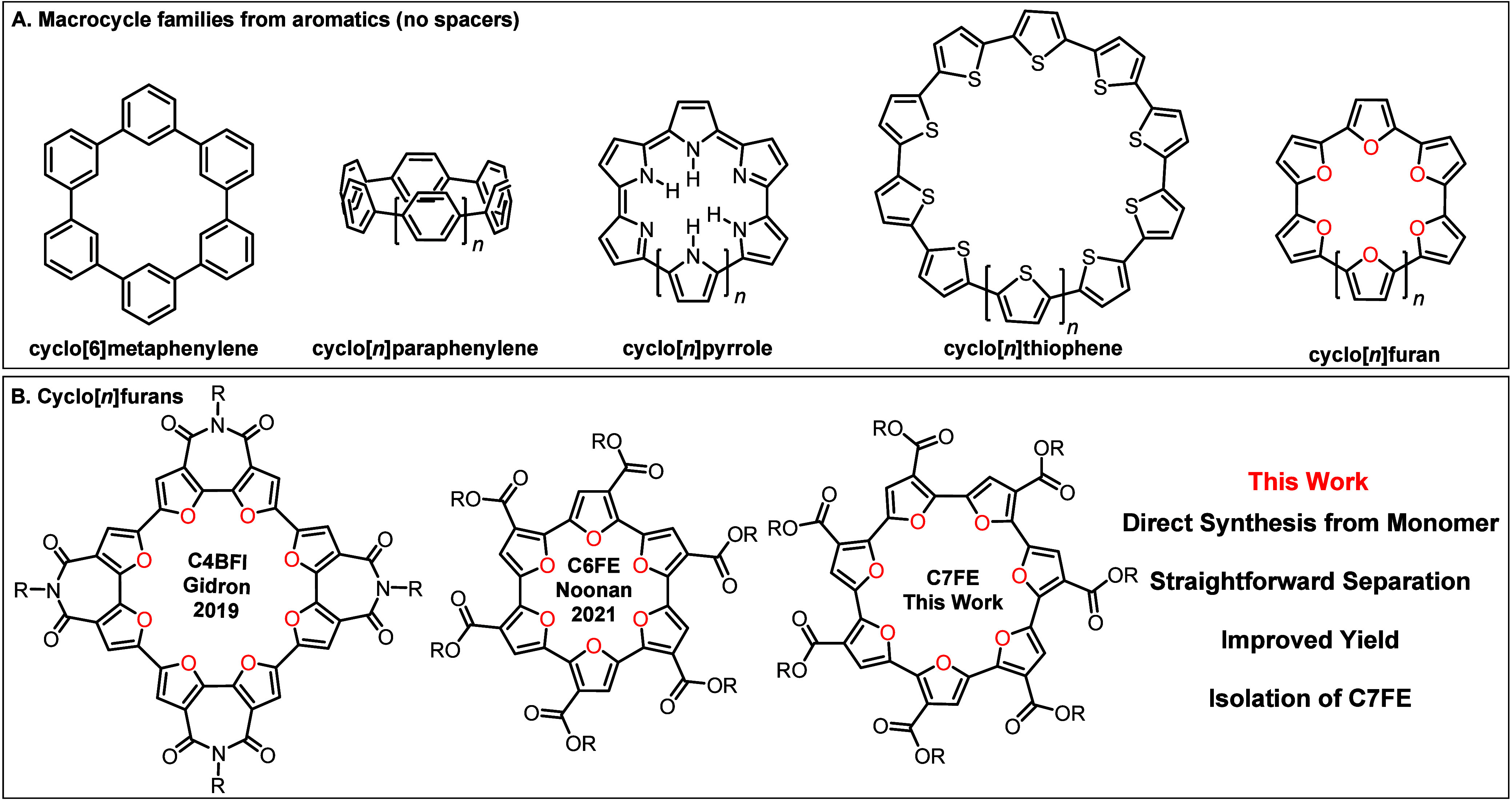
A) Macrocycle families derived from either benzene, pyrrole or thiophene repeat units.^5–11^ B) Recently reported cyclo­[*n*]­furans.^19–22^

Cyclo­[*n*]­furans (CnFs) have been less studied, which may be due to questions regarding the oxidative stability of long chain conjugated oligo- and polyfurans,^23–25^ and the potential of furans to undergo cycloaddition chemistry.^26,27^ Nevertheless, furan is a unique building block for the construction of macrocycles as demonstrated by Gidron and co-workers in a computational study in 2018.^28^ CnFs comprised of 6–8 repeat units were predicted to have the lowest ring strain, which results in planar or near planar macrocycles with all the furan repeat units adopting a *syn* conformation with respect to one another.^28^ This is markedly different than the analogous CnTs, where 11–15 repeat units leads to low strain energies.^18,28^ Moreover, CnTs with 11–15 repeat units are still predicted to be distorted.^18^ The different internal angles for furans versus thiophenes, leads to the prediction of small, compact CnFs,^28^ and the high twist energies around the inter-ring bond in oligofurans,^29,30^ promotes planar or near-planar structures.

To partially combat the oxidative instability of oligofurans, electron-withdrawing groups can be installed on the furan repeat unit, which was critical to the first reports on cyclo­[*n*]­furans.^19–22^ Gidron and co-workers first reported on the cyclo­[*n*]­bifuran diimides in 2019, with recent follow-ups making different sized macrocyclic rings in 2021, and reducing the imide side groups of the bifuran in 2022 (Figure 1 – Bottom).^19–21^ Our group reported on the isolation of a hexyl-cyclo[6]­furan ester (*hex*-C6FE) in 2021, starting from a regioregular ester-substituted furan dimer (Figure 1 – Bottom),^22^ which was obtained from 3-furoic acid in 7% overall yield over the 8 synthetic steps. Alternating furan-acetylene^31^ and furan-thiophene^32^ macrocycles have also been prepared recently.

Herein, we describe a general approach for cyclo­[*n*]­furan ester (CnFE) synthesis, starting from a single furan monomer (Figure 2). In addition, an odd-membered furan macrocycle has been isolated for the first time (*hex*-C7FE). The method enables rapid production of CnFEs from 3-furoic acid in a total of 4 synthetic steps (24% overall yield from the acid). The macrocyclization reaction is carried out like a polymerization reaction, with two key factors playing a role in obtaining >50% conversion to macrocycles. First, the ester side chain on the furan ring leads to an energetic preference for *syn* conformers as the chain length increases (Figure 2). Second, the precise cross-coupling conditions (tri*-tert*-butylphosphine, cesium fluoride and palladium precursor) are also critical to the process. Model compound studies suggest that the reaction proceeds via a catalyst-transfer mechanism,^33–35^ with preferential oxidative addition on the chain-end of the growing oligomer. Protocols to construct precise homocyclic compounds from a single aromatic repeat unit are rare, as conjugated macrocycles are often built via stepwise or iterative coupling. The potential to make precise conjugated macromolecules with specific lengths are highly desirable to control properties and self-assembly. For example, Hawker employed chromatographic separation^36,37^ to create well-defined libraries of discrete conjugated linear oligomers^36^ and Seferos developed a monomer-by-monomer addition reaction to create oligo/polythiophenes of discrete length.^38,39^

**Figure 2:**
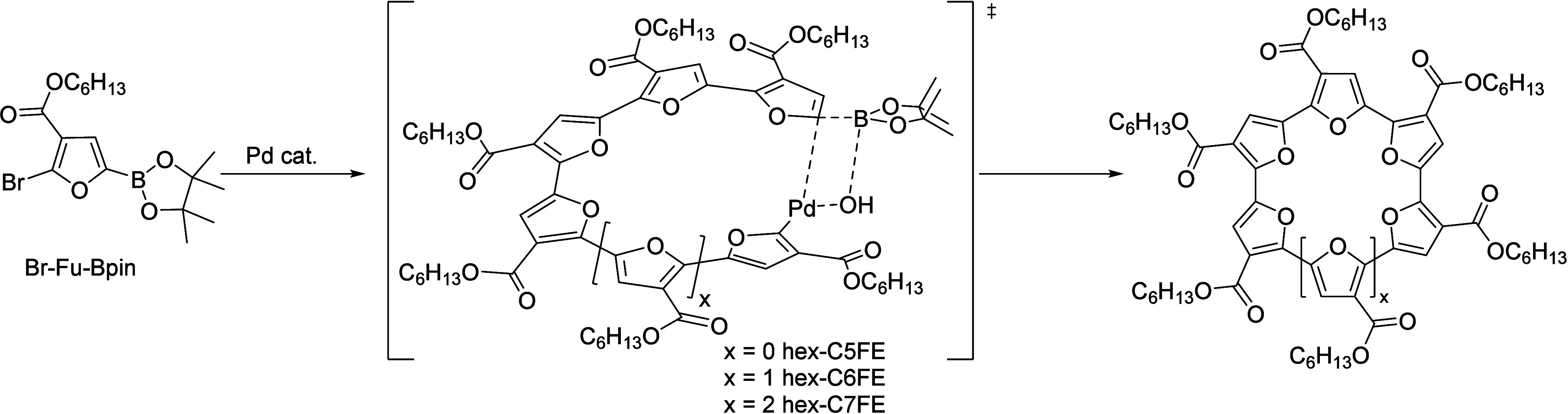
Cyclization concept for a furan monomer using a Pd^0^ catalyst, enabling formation of *hex*-CnFE (where *n* = 5, 6, 7). Figure adapted from ref 22.

## Results and Discussion

### Synthesis

In prior synthetic efforts, we employed the commercially available G3-PdSPhos^40^ to cross-couple hexyl 2-bromo-5-(4,4,5,5-tetramethyl-1,3,2-dioxaborolan-2-yl)­furan-3-carboxylate (Br-Fu-Bpin) and observed 3 macrocyclic products which were difficult to separate (*x* = 0, 1, or 2 in Figure 2).^22^ In addition, low conversion to the three macrocyclic species was observed in the initial study (Table 1, entry 1),^22^ prompting further investigation.

**Table 1:**
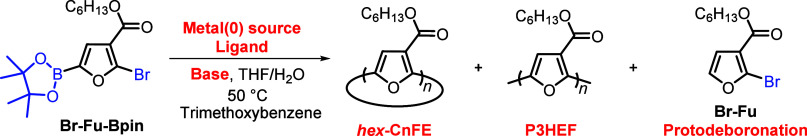
Optimization of Reaction Conditions for Macrocyclization

Entry^a^	Catalyst	Base	Total macrocycle conv.	C5FE	C6FE	C7FE	BrFu
1^b^ ^,^ ^c^	G3PdSPhos	K_3_PO_4_	10	3	6	1	6
2^c^	G3PdSPhos	K_3_PO_4_	21	10	9	2	–
3^c^	G3PdSPhos	CsF	11	1	9	1	22
4	Pd_2_dba_3_ + SPhos	K_3_PO_4_	1	0	1	–	23
5	Pd_2_dba_3_ + SPhos	CsF	4	1	3	0	15
6	Pd_2_dba_3_ + [(*t*-Bu)_3_PH]BF_4_	CsF	52	–	33	19	–
7	Pd_2_dba_3_ + [(*t*-Bu)_3_PH]BF_4_	K_3_PO_4_	4	–	3	1	1
8	Pd_2_dba_3_ + PAd_3_	CsF	51	–	37	14	–
9	Pd_2_dba_3_ + PAd_3_	K_3_PO_4_	1	–	1	0	7
10	Pd_2_dba_3_ + PCy_3_	CsF	12	–	10	2	4
11^c^	G3PdP(*t*-Bu)_3_	K_3_PO_4_	13	–	9	4	–

a Typical conditions: Br-Fu-Bpin monomer (0.125 mmol) was combined with catalyst (10 mol % Pd and 10 mol % ligand), 3 equiv of base, THF:H_2_O (5:1, 30 mM) and trimethoxybenzene (∼0.2 equiv) as an internal standard. Reactions carried out at 50 °C. Conversion was determined using ^1^H NMR spectroscopy after 1 h, and for experiments which lead to >5% conv, the reported values are an average over 3 runs. Concentration of oligomers/polymers (P3HEF) was not quantified.^25^

b THF:H_2_O (30:1) was added.

c 7.7 mol % of the catalyst was added.

The products and side products that can arise with this cross-coupling reaction are shown in the top of Table 1 which includes: formation of *hex*-CnFEs,^22^ formation of open chain oligomers/polymers (P3HEF^25^), and loss of the boron group by protodeboronation (Br-Fu).^41,42^ Formation of the three macrocyclic products (C5FE-C7FE) as well as the protodeboronated species were quantified by crude ^1^H NMR spectroscopy by examination of the aromatic region and comparing to an internal standard (Table 1). Determining conversion to the open chain oligomers and polymers was difficult using this approach since P3HEF has a very broad aromatic signal (spans ∼700 Hz).^25^

Initially, we noted that simply increasing the proportion of water in the cross-coupling reaction had a beneficial effect, nearly doubling the total conversion to 21% for *hex*-C5FE, *hex*-C6FE and *hex*-C7FE using G3PdSPhos (Table 1, entry 2), though effective separation of all 3 macrocycles using chromatography was still difficult. Extensive monomer protodeboronation was observed when G3-PdSPhos was paired with CsF as the base (Table 1, entry 3). The weaker base likely leads to inefficient catalyst activation, so we explored tris­(dibenzylideneacetone)­dipalladium (Pd_2_dba_3_) as a Pd precursor. Surprisingly, the combination of Pd_2_dba_3_, SPhos and a base did not result in significant quantities of CnFE’s formed (Table 1, entries 4–5).

Notably, the combination of tri*-tert*-butylphosphine^43–45^ (P­(*t*-Bu)_3_) and tri­(1-adamantyl)­phosphine^46^ (PAd_3_) with Pd_2_dba_3_ led to >50% conversion to *hex*-C6FE and *hex*-C7FE with CsF (Table 1, entries 6 and 8). A representative stack plot illustrating the conversion determined using ^1^H NMR spectroscopy is shown in Figure 3. *Hex*-C5FE formation is minimal if trialkylphosphine ligands are employed in the macrocyclization (facilitating the separation), and *hex*-C6FE is formed in higher proportion compared to *hex*-C7FE. Attempts to monitor reaction kinetics for the oligomerization were difficult as the Br-Fu-Bpin monomer was typically consumed within 2 min. The air-stable phosphonium precursor ([(*t*-Bu)_3_PH]­BF_4_) was used in all instances to generate P­(*t*-Bu)_3_ *in situ*, simply due to ease of handling and air stability as noted by Fu and co-workers previously.^47^ Since PAd_3_ has been reported to be an air-stable crystalline solid,^46^ this was used directly in that form. When using K_3_PO_4_ as the base, the monomer is still consumed rapidly, but CnFEs are formed in very small proportions (Table 1, entries 7, 9 and 11). These results highlight the critical importance of CsF as the base for cyclization.

**Figure 3:**
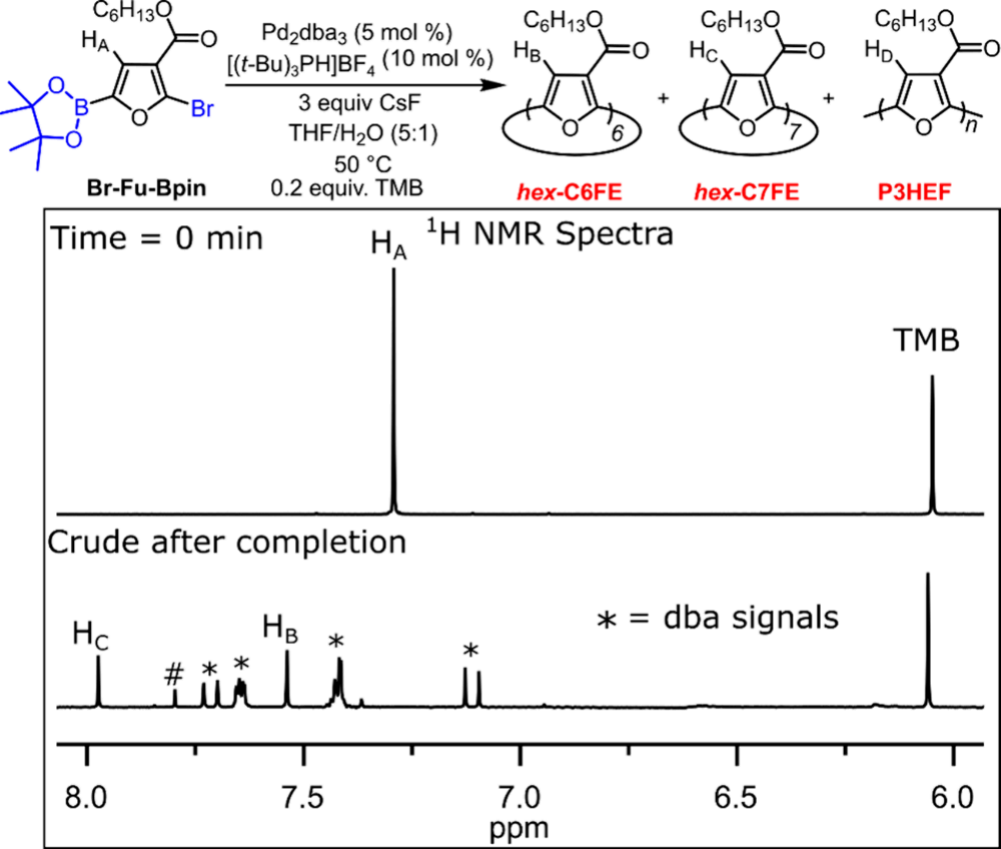
Stack plot of the crude ^1^H NMR spectra (500 MHz, CD_2_Cl_2_, 22 °C) collected for a macrocyclization experiment with Pd_2_dba_3_ (5 mol %), [(*t*-Bu)_3_PH]­BF_4_ (10 mol %) and CsF (3 equiv). Conversion to macrocycle was determined by comparison with the aromatic signal from the 1,3,5-trimethoxybenzene (TMB) internal standard (0.2 equiv). Top corresponds to *t* = 0 min and monomer (H_A_). Bottom corresponds to *t* = 1 h after addition of catalyst, base and water. New signals for *hex*-C6FE (H_B_) and *hex*-C7FE (H_C_). The * signals correspond to the dba ligand from the catalyst and the # signal is unidentified.

Ananikov and co-workers have noted that Pd_2_dba_3_ decomposes over time, and that the degree of purity can vary depending on commercial sources.^48^ Our findings are consistent with that report, and macrocycle conversion dropped to ∼20% when utilizing a commercial sample of 40% pure Pd_2_dba_3_ (Figure S5). Given this, the precatalyst was purified via recrystallization in CHCl_3_ prior to use in macrocyclizations (Figure S4).^48^

The fast reaction time and good yield for the cyclization suggested that this reaction may proceed by a different mechanism than the expected step-growth process. Chain-growth polycondensation of aromatics to form conjugated polymers has been hypothesized to proceed by association of the catalyst to the growing chain during the reaction via a π-bonding interaction between the metal and growing chain.^33–35^ Direct evidence to illustrate that the metal coordinates to the growing aromatic chain is rare,^49^ while indirect evidence for this process has been commonly inferred from model reactions.^50–55^ If aromatic dihalides are combined with a stoichiometric deficiency of a cross-coupling partner and only undergo exhaustive coupling to form trimers (with leftover starting material remaining in the reaction mixture), this suggests that the catalyst remains coordinated to the aromatic substrate after the first coupling and “walks”^56^ to the other halide for subsequent intramolecular activation.^50–55^ A model reaction was examined to provide evidence for a chain-growth mechanism (Table S2), which we hypothesize contributes to the fast reaction rate and improved yield in this case.

Ethyl-2,5-dibromofuran-3-carboxylate was combined with furan-2-boronic acid pinacol ester (FuBpin) in a 2:1 ratio with a small selection of catalysts (Table S2). For G3PdSPhos and G3PdP­(*t*-Bu)_3_ complete consumption of the boronic ester was observed with 94% and 99% conversion to trimer, respectively (Table S2, entries 1–2). This suggests that the Pd P­(*t*-Bu)_3_ may be slightly more efficient in the chain-growth process for the oligomerization of the furan monomer. We also carried out the same cross-coupling reaction with Pd_2_dba_3_ and the 3 different trialkylphosphines (Table S2, entries 3–5). As expected, both P­(*t*-Bu)_3_ and PAd_3_ afforded >98% conversion to the furan trimer, in line with the good yields for macrocycle formation.

### Spectroscopic Properties

The separation of *hex*-C6FE and *hex*-C7FE from P3HEF was accomplished using column chromatography (Supporting Information). Minor impurities attributed to the open chain oligomers were removed by washing the solid with acetonitrile, followed by recrystallization via solvent diffusion using CH_2_Cl_2_ and methanol (1:3). The purity of *hex*-C6FE and *hex*-C7FE was confirmed by NMR spectroscopy, MALDI-TOF mass spectrometry, and FTIR spectroscopy (Figure 4A-D) and the spectral data for *hex*-C6FE was consistent with the prior report.^22^ A sharp singlet corresponding to the aromatic proton for *hex*-C6FE is observed at 7.46 ppm (H_D_ in blue, Figure 4A), while the aromatic signal for *hex*-C7FE appears ∼0.5 ppm further downfield at 7.99 ppm (H_D_ in red, Figure 4A). It should be noted that the aromatic signal for *hex*-C6FE can range from δ ∼ 7.60–7.45 ppm depending on concentration in CDCl_3_, due to aggregation.^22^ The triplet signal corresponding to the methylene protons of the hexyl ester side chain appears at 4.28 ppm for *hex*-C7FE (H_F_ in red, Figure 4A), 0.11 ppm downfield as compared to the same signal for *hex*-C6FE (4.17 ppm, H_F_ in blue, Figure 4A). The other signals for the alkyl groups (0.89 ppm to 1.80 ppm) also exhibit slight downfield shifts in *hex*-C7FE compared to those in *hex*-C6FE.

**Figure 4:**
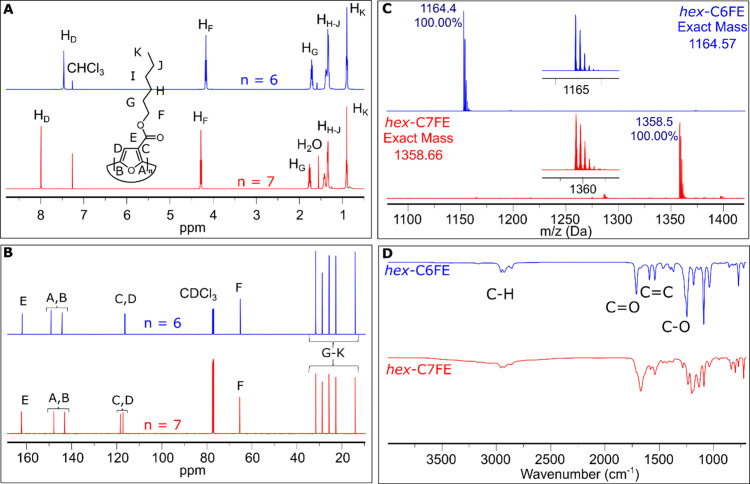
(A) Stack plot of the ^1^H NMR spectra (500 MHz, CDCl_3_, 22 °C) of *hex*-C6FE (Blue) and *hex*-C7FE (Red). (B) Stack plot of the ^13^C­{^1^H} NMR spectra (126 MHz, CDCl_3_, 22 °C) of *hex*-C6FE (Blue) and *hex*-C7FE (Red). (C) MALDI-TOF mass spectra of *hex*-C6FE (Blue) and *hex*-C7FE (Red). (D) FT-IR (ATR) spectra of *hex*-C6FE (Blue) and *hex*-C7FE (Red). The NMR and MALDI-TOF data for *hex*-C6FE were previously reported,^22^ and are included for comparison.

The ^13^C­{^1^H} NMR spectra of the two different CnFE macrocycles are similar, though some subtle key differences are observed. Specifically, the C2 and C5 carbons of the furan ring (labeled A and B in Figure 4B) are upfield by ∼1 ppm for *hex*-C7FE. Furthermore, while the C3 and C4 carbons of the *hex*-C6FE ring are nearly overlapping at 116.4 and 116.3 ppm, while these are much more distinct in *hex*-C7FE at 118.4 and 117.3 ppm (labeled C and D in Figure 4B).

The progressive downfield shift for the aromatic signal in the ^1^H NMR spectra of the *hex*-CnFEs is quite different than in the previously reported cyclo­[*n*]­thiophenes, where ^1^H NMR chemical shifts change minimally with larger ring size.^11^ To explore the chemical shift differences in more detail, density functional theory (DFT) calculations of NMR shielding constants were computed using the gauge independent atomic orbital (GIAO) method. The predicted spectra are shown in the Supporting Information (Figures S36 and S37). Geometry optimizations were completed on cyclo[6]- and cyclo[7]­furans with and without side chains for comparison. Optimizations were carried out in all instances using the B3LYP-D3­(BJ)^57^ functional and a 6-31G­(d,p) basis set, with a continuum solvation model (IEFPCM) in CH_2_Cl_2_.

The parent 6- and 7-membered macrocycles (no alkyl or ester substituents) are denoted as C6F and C7F (structure shown in Figure S36). Macrocycles with methyl groups on each furan repeat unit are denoted as *me*-C6F and *me*-C7F (structure shown in Figure S36). Finally, macrocycles with the methyl ester groups on each furan are denoted as *me*-C6FE and *me*-C7FE (direct analogs of the synthesized *hex*-C6FE and *hex*-C7FE). The chemical shift of the aromatic signals are predicted to be the same for C6F and C7F (7.5 ppm) and the methyl-substituted derivatives *me*-C6F and *me*-C7F (7.3 ppm). In contrast, a large downfield chemical shift is predicted with the larger macrocycle if ester side chains are present on the furan rings (δ ∼ 9.5 and 10.5 ppm predicted for the aromatic signals of *me*-C6FE and *me*-C7FE, respectively). A shortened distance (and stronger interaction) between the carbonyl oxygen and adjacent aromatic furan ring proton (2.27 Å in *me*-C6FE to 2.10 Å in *me*-C7FE) was noted in the computed structures. This decreased distance is likely the main contributor to the observed downfield chemical shift. The NMR-GIAO calculations are also consistent with the changes observed in the ^13^C­{^1^H} spectrum (Figure S37).

The major peak observed in MALDI-TOF mass spectrometry for each macrocycle is a close match to the expected mass: 1164.4 g/mol for *hex*-C6FE (blue) and 1358.5 g/mol for *hex*-C7FE (red) as shown in Figure 4C. The isotope patterns also match with expectation for both *hex*-C6FE and *hex*-C7FE (Figure 4C) further confirming the successful formation and isolation of the macrocycles. The FTIR spectra of *hex*-C7FE and *hex*-C6FE are similar, but the 7-membered macrocycle spectrum is clearly broadened, with slightly shifted absorption bands (Figure 4D). The C=O stretching frequency in *hex*-C7FE is broader (∼1670 cm^–1^), slightly weaker, and merges with C=C vibrational modes when compared to the C=O stretch for *hex*-C6FE (1709 cm^–1^). Additionally, the C–O stretching differs in intensity and is broader in *hex*-C7FE. These spectral differences suggest variations in bond environments, likely influenced by ring strain and conformational effects. To further investigate these factors, we analyzed ring strain in both macrocycles and examined their flexibility using molecular dynamics simulations.

### Computed Ring Strain

The geometry optimizations for *me*-C6FE and *me*-C7FE revealed the ester-functionalized 7-membered ring is slightly curved and likely to be more strained (Figure 5). The open form of the 7-mer (*me*-L7FE) shows partial overlap of the furan end groups, while the 6-mer (*me*-L6FE) does not (Figure 5A). Indeed, the computed ring strain energy for *me*-C7FE is 14.9 kcal/mol while that of *me*-C6FE is 3.1 kcal/mol, suggesting decreased strain for the 6-mer. The ring strain energies were computed using a hyperhomodesmotic^58^ reaction equation (Figures S29 and S30). The side chain substituents also impact the computed strain energies for the macrocycles as shown in the Supporting Information (Figures S29–S34). For example, the computed strain for C6F (6.4 kcal/mol) and C7F (2.4 kcal/mol) suggests that formation of the 7-mer is more favorable without ester groups present (when compared to the all *syn* open-chain form). It should also be noted a range of approaches exist to compute strain,^58^ which will impact the final values.

**Figure 5:**
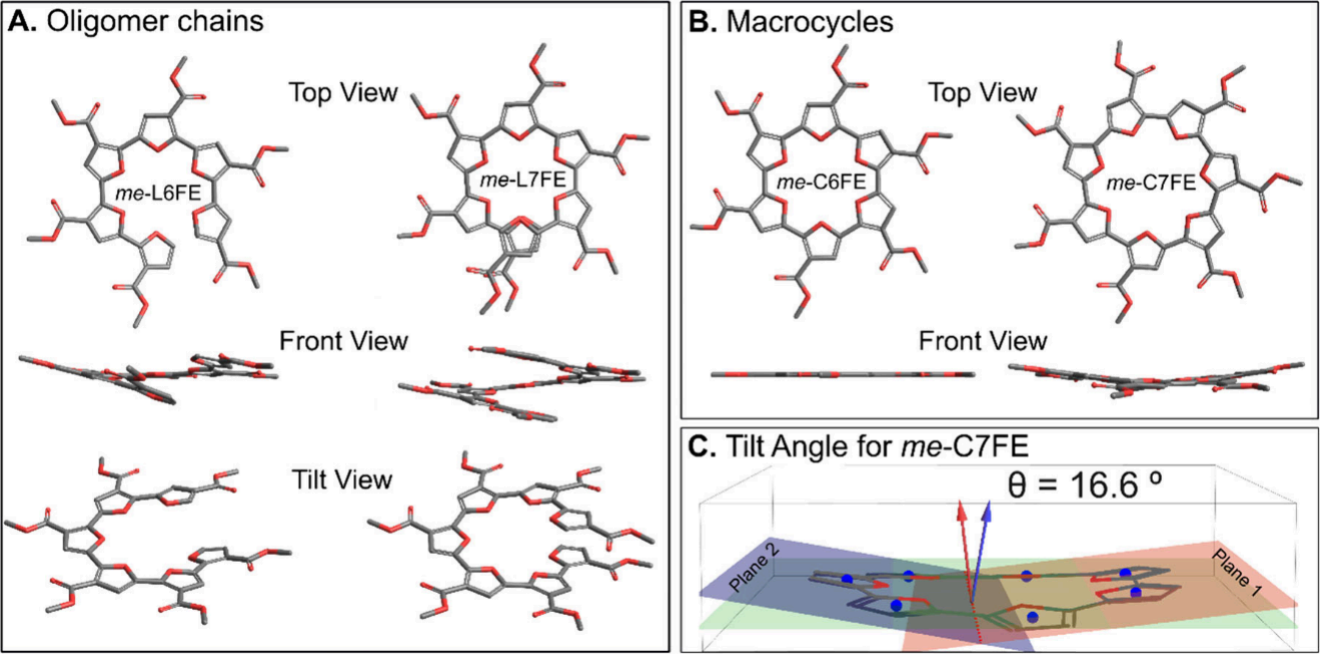
(A) Optimized structures of open furan ester oligomers from DFT calculations at the B3LYP-D3­(BJ)/6-31G­(d,p) level of theory with a continuum solvation model (IEFPCM) in CH_2_Cl_2_. (B) Optimized structures of cyclized furan ester oligomers, computed as noted in (A). (C) The dihedral angle (θ) between the planes formed by the *me*-C7FE (plane 1, red; plane 2, blue), calculated from the optimized structure. Side chains are omitted for better visualization.

### Molecular Dynamics

We then considered whether the higher ring strain and curved geometry for *hex*-C7FE might impact the conformational flexibility of the structure. Molecular dynamics (MD) simulations were carried out using GFN2-xTB on *me*-C6FE and *me*-C7FE.^59–61^ This semiempirical approach significantly reduces the computational cost of molecular simulations but retains essential quantum mechanical features, which makes it a useful tool for large molecular systems. The parametrization of GFN2-xTB employs global and element-specific parameters, which ensures a consistent description across different chemical environments without relying on specific pairwise parameters.^60^ This characteristic is particularly advantageous when analyzing molecular flexibility, as it allows for a comprehensive sampling of possible conformations without the need for highly customized parameter sets. The MD simulations were performed with an initial heating phase to bring the system to the desired temperature, followed by equilibration and production phases. During the production phase, the structures were sampled at regular intervals to capture the range of possible conformations.

Simulations were carried from 300 to 500 K with steps of 50K. Trajectories were obtained for 100 ps, and sampling was performed every 1 fs. The SHAKE algorithm,^62^ which constrains all bonds during simulation (no bond breaking), was turned off to capture C–H stretching vibrations. The dihedral angles of rotatable C–O bonds in the computed *me*-C6FE and *me*-C7FE structures (highlighted in Figure 6) were extracted along the trajectory of the molecular dynamics’ simulation, to analyze flexibility. The changes in these torsional angles are periodic, which enabled a Fourier Transform of the peaks in the trajectory to obtain vibration related frequencies.

**Figure 6:**
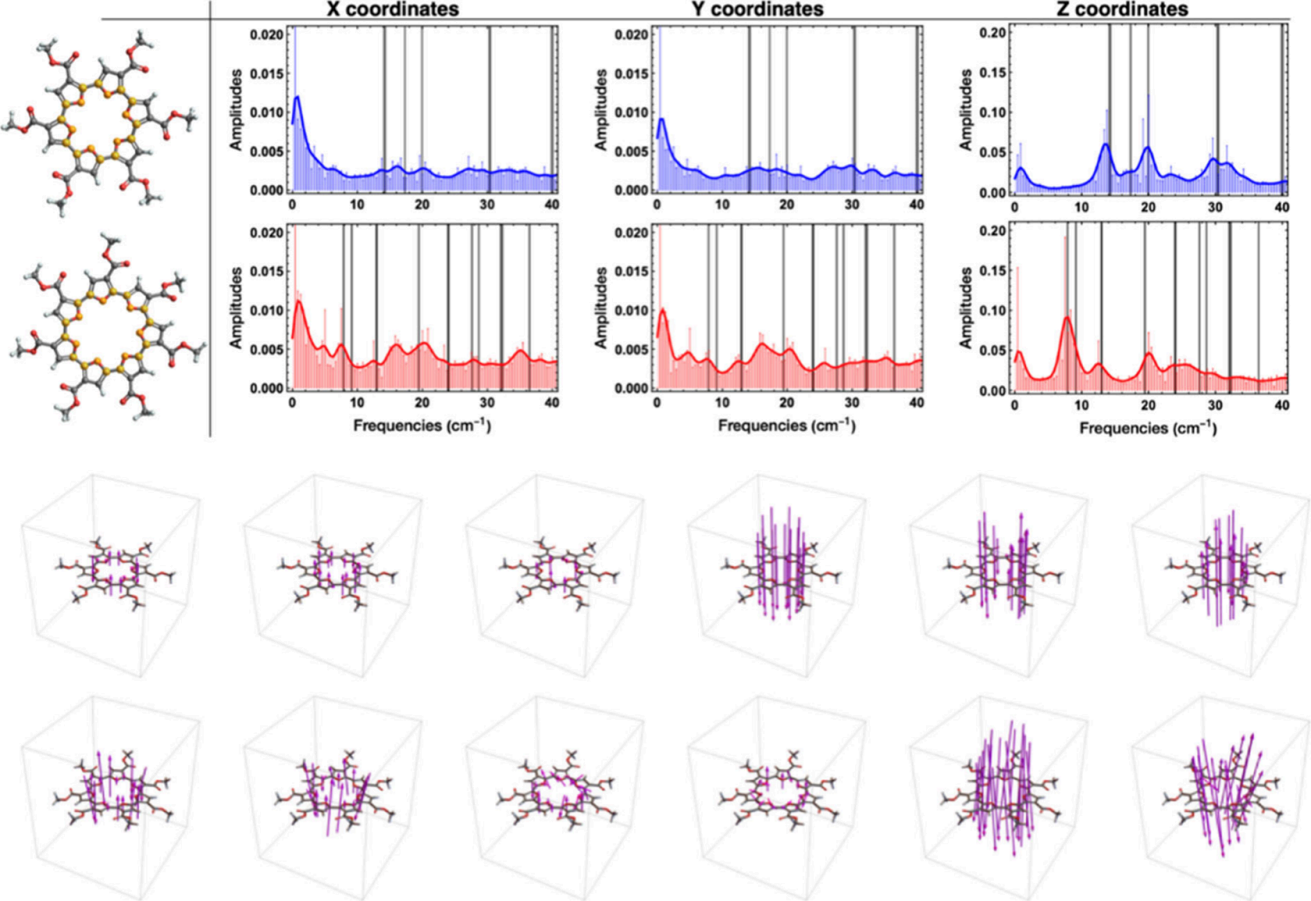
Top left - dihedral angles of rotatable C–O bonds in *me*-C6FE (top) and *me*-C7FE. Top right - low vibrational frequencies of *me*-C6FE (blue) and *me*-C7FE (red) obtained from molecular dynamics. The black lines represent normal modes calculated by xTB from DFT optimized geometries. At the bottom of the scheme, pink vectors illustrate the 6 lowest normal modes calculated by xTB for both *me*-C6FE and *me*-C7FE.

Figure 6 also illustrates the computed low-frequency vibrational modes of the *me*-C6FE (blue) and *me*-C7FE (red) macrocycles from molecular dynamics simulations,^63,64^ alongside the normal modes calculated by xTB (black lines).^59^ These frequencies, found at the low-energy end of the vibrational spectrum, correspond to collective deformations of the macrocyclic framework, including torsional, bending, and puckering motions. The pink vectors shown on the structures (Figure 6) illustrate the atomic displacements associated with the six lowest normal modes, as calculated by xTB. The presence of distinct peaks and their relative intensities reflect the inherent flexibility of each macrocycle, highlighting how ring size and topology influence the accessible conformational landscape.

The larger number of low-frequency modes observed for *me*-C7FE as compared to *me*-C6FE (from both molecular dynamics and xTB), is consistent with increased conformational freedom arising from its larger size. The six-membered system displays relatively well-defined low-frequency modes, suggesting a more restricted dynamic range of accessible conformations. The seven-membered ring supports a broader set of low-energy deformations with the additional peaks in the low-frequency region, indicating the presence of multiple modes associated with subtle ring distortions and torsion. As a result, the seven-membered macrocycle can more readily transition between various geometries with lower energy barriers.

### Optical and Redox Properties

The UV–vis spectrum of *hex*-C7FE was collected and compared with *hex*-C6FE (Figure 7A-B). The λ_max_ for *hex*-C7FE is red-shifted by 17 nm relative to *hex*-C6FE, which is expected due to the additional furan repeat unit. Time-dependent DFT (TD-DFT) calculations on *me*-C7FE (dotted red trace, Figure 7B) align with experimental data, predicting an intense S_0_ → S_2_/S_3_ transition at 373 nm, 25 nm red-shifted compared to TD-DFT results for *me*-C6FE. The computed S_0_ → S_2_ and S_0_ → S_3_ transitions occur at the same wavelength and oscillator strength for each macrocycle due to the double degeneracy of the higher-energy unoccupied molecular orbitals following the singly degenerate LUMO. The HOMO–LUMO transition for both *me*-C6FE and *me*-C7FE is predicted to have zero oscillator strength (*f* = 0). The symmetry-forbidden *S*_0_→*S*_1_ transition for *hex*-C7FE occurs at ∼510 nm, close to the predicted transition at 534 nm. Notably, the absorption profile of *hex*-C7FE lacks the distinct structure seen in *hex*-C6FE, which is consistent with the prediction that it is more conformationally flexible. The featureless absorption profile is similar to the alternating ester-functionalized furan-thiophene macrocycle as well as the cyclo[10]­thiophene.^15,32^ The decreased rigidity for *hex*-C7FE may also partially explain the slightly increased intensity for the formally forbidden HOMO–LUMO transition (∼510 nm). No significant luminescence was noted for either macrocycle in solution.

**Figure 7:**
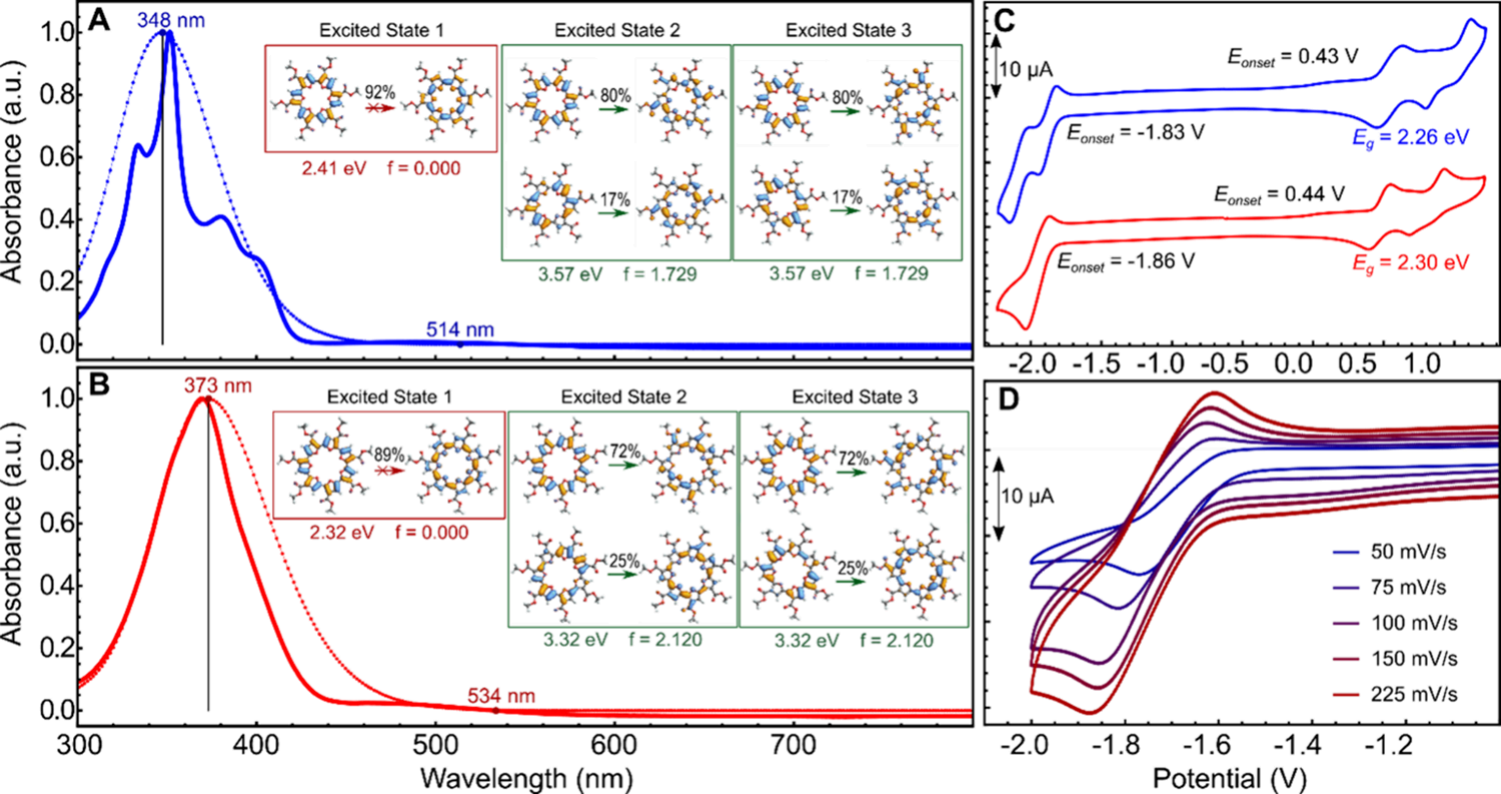
Normalized absorption spectrum of *hex*-C6FE (A) and *hex*-C7FE (B) collected in CHCl_3_ (solid line) and predicted by TD-DFT (dotted line, CAM-B3LYP/6–31G­(d,p) IEFPCM­(CH_2_Cl_2_)). The calculated natural transition orbitals are shown in the inset. (C) Cyclic voltammograms of *hex*-C6FE (blue trace) and *hex*-C7FE (red trace) in degassed CH_2_Cl_2_ (0.63 mg/mL) using NBu_4_PF_6_ as the supporting electrolyte (0.07 M), with a scan rate of 100 mV/s. Voltammograms were referenced using Fc/Fc^+^ as an internal standard.^66^ Determination of the electrochemical band gap of *hex*-C7FE from the onsets of oxidation and reduction at +0.44 and −1.86 V, respectively, yields a value of *E*_*g*_ = 2.30 eV. (D) Overlay of the cyclic voltammograms of *hex*-C7FE with varying scan rates in degassed CH_2_Cl_2_ (0.63 mg/mL) using NBu_4_PF_6_ as the supporting electrolyte (0.07 M).

Cyclic voltammetry studies of *hex*-C7FE were carried out in CH_2_Cl_2_ with Fc/Fc^+^ with NBu_4_PF_6_ as the supporting electrolyte (Figure 7C). Half-wave potentials are reported for the quasi-reversible oxidation and reduction while the inflection potential (*E*_i_) is reported to estimate the potential for the second oxidation.^65^ Quasi-reversible oxidations were observed at 0.49 V (*E*_1/2_) and 0.91 V (*E*_i_) for *hex*-C7FE (0.56 and 1.09 V for *hex*-C6FE). Both oxidations are lower for *hex*-C7FE, reflecting the influence of the extra repeat unit. A single quasi-reversible reduction is observed for *hex*-C7FE (*E*_1/2_ = −1.96 V), which is more cathodic than the first of two quasi-reversible reductions observed for *hex*-C6FE (*E*_1/2_ = −1.89 V and −2.09 V). Given the additional current passed for reduction as compared to oxidation, it is possible that the reduction is a 2-electron process for *hex*-C7FE. Notably, the quasi-reversible reduction for *hex*-C7FE became irreversible at low scan rates (50 mV/s) and more reversible at faster scan rates (225 mV/s in Figure 7D). This irreversibility at lower scan rates suggests instability of the reduced species, which was also noted for P3HEF previously.^25^ Both macrocycles exhibit reasonable redox stability, as repeated cycling at the same scan rate produces essentially identical voltammograms.

Optimizations and frequency calculations to calculate ionization potentials (IP) and electron affinities (EA) for the first and second redox steps of *me*-C6FE and *me*-C7FE were carried out at the B3LYP-D3­(BJ)/6-31G­(d,p) levels using a continuum solvation model (IEFPCM) with CH_2_Cl_2_ as the solvent (Figure S35).^32^ The first oxidation and first reduction were used to estimate the computed bandgap (2.15 and 1.96 eV for *me*-C6FE and *me*-C7FE, respectively). The electrochemical bandgap (*E*_g_), estimated from oxidation and reduction onsets, was within 0.11 eV of the computed gap for *hex*-C6FE (2.26 eV). A larger deviation (0.34 eV) was noted for *hex*-C7FE (*E*_g_ = 2.3 eV). The optical and electrochemical bandgaps are in a similar range for both macrocycles, but it should be noted that the optical gap is slightly lower for *hex*-C7FE while the electrochemical gap is slightly lower for *hex*-C6FE.

While the neutral form of *me*-C7FE is predicted to be slightly curved, the doubly oxidized and reduced forms are predicted to be planar as shown in Figure 8. Nucleus Independent Chemical Shift (NICS)^67^ was used to determine whether the macrocycle is globally aromatic upon double oxidation or reduction, similar to prior work.^22,32^ The zz components of NICS were computed at 1 Å above the plane of the macrocycle and values are reported in ppm and shown in Figure 8 (NICS(1)_*zz*_). Both the doubly reduced and doubly oxidized have large negative NICS(1)_*zz*_ values (−21.7 and −33.8), indicating a diatropic ring current and an aromatic structure. This prediction for *me*-C7FE is also consistent with the prior results for *me*-C6FE and the 8-membered ester-functionalized alternating thiophene-furan macrocycle.^22,32^

**Figure 8:**
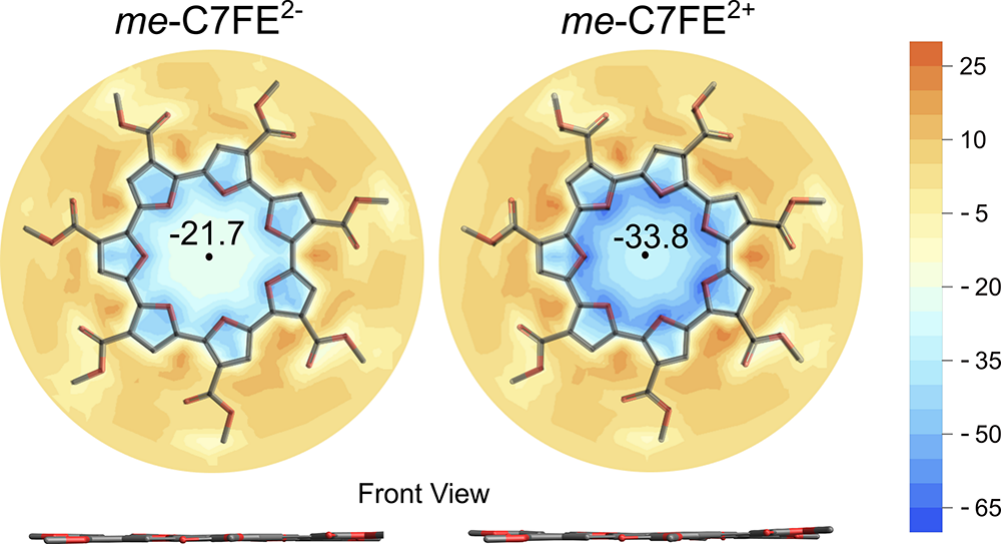
NICS­(1)_*zz*_ plots for *me*-C7FE in their dianion (left) and dication (right) state. The color scale bar indicates the degree of aromaticity or antiaromaticity, with more negative values representing a higher aromatic character and positive values representing greater antiaromatic character. Macrocycles are annotated with NICS values at the center of the cycle. Calculations were performed using the NMR-GIAO method with the B3LYP-D3­(BJ) functional and 6-31G­(d,p) basis set, employing a continuum solvation model (IEFPCM) in CH_2_Cl_2_. NICS values were obtained on a polar grid placed 1 Å above the macrocycle plane.

## Conclusion

In this work, we have demonstrated that hexyl 2-bromo-5-(boronic acid pinacol ester)­furan-3-carboxylate can be converted to a cyclo­[*n*]­furan (where *n* = 6 or 7) in good yield in the presence of Pd_2_dba_3_, P­(*t*-Bu)_3_ and CsF. The rapid reaction kinetics, along with model studies, suggest that the process may proceed via a chain-growth mechanism. An odd-membered hexyl-substituted cyclo[7]­furan ester was isolated for the first time, and the optical and redox properties of this structure were examined. Spectroscopic and computational studies revealed that the ester-functionalized cyclo[7]­furan is more strained, slightly curved and more conformationally flexible than the 6-membered analog. Computational studies predict the 7-membered macrocycle is planar and globally aromatic in the doubly oxidized and reduced states, like its 6-membered counterpart. Altogether, the developed synthetic method offers straightforward access to cyclo­[n]­furans of different sizes, and future work will focus on synthetic modifications to the macrocycle, metal coordination and examination of molecular packing in the solid-state.

## Supplementary Material







## Data Availability

The data underlying this study are available in the published article and its .

## Notes

Experimental procedures, NMR spectra, gas chromatograms (GC), MALDI-TOF mass spectra, absorbance spectra, cyclic voltammograms, computed energies, computed NMR spectra, computed orbital energies (PDF)
FAIR data, including the primary NMR FID files, for compounds *hex*-C6FE and *hex*-C7FE (ZIP)
Optimized geometries of computed structures in xyz format (ZIP)
