# Supplementary material for: Precise Synthesis of Ester-Functionalized Cyclo[6]- and Cyclo[7]furans
Source: J Org Chem. 2025 Jul 7;90(28):9733–42. doi: 10.1021/acs.joc.5c00526 (PMC12313054; doi:10.1021/acs.joc.5c00526)
Supplement: Supplementary file 1 [file jo5c00526_si_001.pdf]

Supporting Information

Precise Synthesis of Ester-Functionalized Cyclo[6]- and Cyclo[7]furans

Dhruv Sharma, Leticia Maria Pequeno Madureira, Tomasz Kowalewski and Kevin J. T. Noonan**

Department of Chemistry, Carnegie Mellon University, 4400 Fifth Ave, Pittsburgh, Pennsylvania, 15213, United States

Table of Contents

Materials and Methods S3-5

Experimental Procedures for Macrocycle Synthesis and Model Studies S6-11

- **Scheme S1.** Synthesis of *hex*-C6FE and *hex*-C7FE S6
- **Figure S1.** Chromatographic separation and TLC analysis of *hex*-C6FE and *hex*-C7FE ... S9
- **Scheme S2.** Model studies with ethyl-2,5-dibromofuran-3-carboxylate S10

NMR Spectra S12-26

- **Figures S2 – S3.** ^{1}H and ^{13}C NMR spectra of *hex*-C6FE and *hex*-C7FE S12-13
- **Figure S4.** ^{1}H NMR spectra of commercial and recrystallized Pd_2dba_3 .... S14
- **Figure S5.** Crude ^{1}H NMR spectra of macrocyclization using 40% pure Pd_2dba_3 .... S15
- **Figures S6 – S16.** Representative crude ^{1}H NMR spectra of the macrocyclization reactions corresponding to the results from Table 1. .... S16-26

GC-MS chromatograms for the model studies in Table 2 **(Figures S17 – S21)**...................... S27-29

MALDI-TOF mass spectra of *hex*-C6FE and *hex*-C7FE **(Figures S22 – S23)**............................ S30

Model Compound Reactions with Ethyl-2,5-dibromofuran-3-carboxylate **(Table S2)**.............. S31

Absorbance spectra of *hex*-C7FE and plot of Abs versus Conc **(Figure S24)**.............................. S32

Cyclic voltammograms and onsets for *hex*-C6FE and *hex*-C7FE **(Figures S25 – S28)**.........S33-36

Computed ring strain energies for *me*-CnFE, CnF, and *me*-CnF **(Figures S28 – S34)** S37-38

Computed Gibbs free energies, IPs, EAs for *me*-C6FE, and *me*-C7FE **(Figure S35)**................ S39

Computed NMR spectra of C6F, C7F, *me*-C6F, and *me*-C7F **(Figure S36)**................................ S40

Computed NMR spectra of *me*-C6FE, and *me*-C7FE **(Figure S37)**... S41

References S42-43

Materials and Methods. All reactions and manipulations of air and water-sensitive compounds were carried out under a dry N_2 atmosphere using a mBraun glovebox or standard Schlenk techniques with dried and degassed solvents. All reagents were purchased from commercial sources and used as received. Solvents and chemicals used for extraction and column chromatography were used as received. Hexyl 2-bromo-5-(4,4,5,5-tetramethyl-1,3,2-dioxaborolan-2-yl)furan-3-carboxylate,[1] and (tri-*tert*-butylphosphine)[2-(2′-amino-1,1′-biphenyl)]palladium(II) methanesulfonate[2] (G3PdP(*t*-Bu)$_3$) were prepared according to literature procedures. The Pd_2dba_3 complex, received at 40% purity, was purified by recrystallization to (>95% purity), yielding crystalline black needles (Figure S4). The purity of Pd_2dba_3 was determined following a method reported in the literature,[3] and the purification was performed using the recrystallization procedure described in the same report.

Flash chromatography was completed using a Biotage Isolera One Flash Chromatography System with Aldrich technical grade silica gel (pore size 60 Å, 70-230 mesh, 63-200 μm).

NMR Analysis. All NMR spectra were recorded at 300 K on either a 500 MHz Bruker Avance 3 Spectrometer or a 500 MHz Bruker Neo Spectrometer with Prodigy Cryoprobe (126 MHz for $^{13}C\{^1H\}$). The ^1H NMR spectra are referenced to $CHCl_3$ (7.26 ppm) and $CHDCl_2$ (5.32 ppm) and the $^{13}C\{^1H\}$ NMR spectra are referenced to $CDCl_3$ (77.2 ppm).

Mass Spectrometry. MALDI-TOF measurements were performed on a Bruker UltraFlextreme MALDI-TOF-MS in linear mode with *trans*-2-[3-(4-*tert*-butylphenyl)-2-methyl-2-propenylidene]malononitrile (DCTB) as the matrix. A 10 mg/mL DCTB solution in $CHCl_3$ was drop-cast onto the target plate. Subsequently, a 20 mg/mL macrocycle solution in $CHCl_3$ was mixed with the matrix on the same target plate.

GC-MS Analysis. GC-MS analysis was performed on an Agilent 6890-5973 GC-MS workstation. The GC column was a Hewlett-Packard fused silica capillary column crosslinked with 5% phenylmethylsiloxane. Helium was used as the carrier gas. The following conditions were used for all GC-MS analyses: injector temperature, 250 °C; initial temperature, 70 °C; temperature ramp, 10 °C/min; final temperature, 290 °C. Sample solution was filtered through a 0.22 µm PTFE syringe filter into a 2 mL vial and diethyl ether was added to fill the vial.

Infrared Spectroscopy. Fourier transform infrared (FTIR) attenuated total reflectance spectroscopy was performed using a PerkinElmer Frontier FTIR spectrometer with a germanium crystal. Spectra were acquired with a 4 cm^{-1} resolution over 700-4000 cm^{-1}.

Cyclic Voltammetry. Electrochemical potentials were determined using a Bio-Logic SP-150 potentiostat. Electrodes were polished using diamond paste prior to running an experiment. A 1 mm^2 glassy carbon working electrode, a platinum coil counter electrode, and a silver wire pseudo-reference electrode were employed for the measurements. The voltammograms were referenced using Fc/Fc^+ as an internal standard. Tetra-*n*-butylammonium hexafluorophosphate was used as the supporting electrolyte at a concentration of 0.07 M in CH_2Cl_2. The CH_2Cl_2 solutions with the supporting electrolyte and analyte were degassed for 10 minutes with argon bubbling before measurement. Cyclic voltammograms are plotted using the IUPAC convention.The initial potential was ~ –0.6 V (versus Fc/Fc^+), and scans were initially swept to positive potentials (oxidative). The total range for the sweep was –2.25 V to 1.25 V (versus Fc/Fc^+).

UV-Vis Spectroscopy. UV-Vis spectra of both macrocycles were recorded on an Agilent 8453 spectrophotometer at 298 K. Before recording the spectra, a 100% transmittance sample of the

cuvette (quartz, 10 mm × 10 mm) was taken. The “blank” of the solvent ($CHCl_3$) was then collected for baseline subtraction during analysis. Solution measurements were completed using $CHCl_3$ as the solvent.

Computational Studies. Density functional theory (DFT), time-dependent DFT (TD-DFT), and nucleus-independent chemical shift (NICS) calculations were performed with Gaussian 16.[4] Geometry optimizations for all compounds were performed at the B3LYP-D3(BJ)/6-31G(d,p) level.[4] TD-DFT calculations were performed at the CAM-B3LYP 6-31G(d,p) level using the Polarizable Continuum Model (PCM) using the integral equation formalism variant (IEFPCM) and CH_2Cl_2 as the solvent. Natural Bond Orbital calculations were performed using the NBO 7 package.[5] Natural transition orbital (NTO) analyses were carried out using the Multiwfn[6] package with results from DFT (NBO) or TD-DFT (NTO) calculations. Isosurface images were generated in Multiwfn[6] or using custom routines written in Mathematica (Wolfram Research, Inc). NICS[7] calculations were performed using the Gauge-Independent Atomic Orbital (NMR-GIAO) method at the B3LYP-D3(BJ)/6-31G(d,p) level on a polar grid of points placed 1 angstrom above the plane of the macrocycle. The results were visualized using Mathematica with custom-written routines.

Optimized geometries for all relevant structures are included as a compressed folder with relevant .xyz files. The naming scheme follows the form: (C/L)#F(E/M/H)-(q)-S/T_b, where # corresponds to the number of repeat units and optional characters shown in parentheses indicate the molecule form (C – cyclic, L – uncyclized oligomer), the side group (E – ester functionality, M – methyl, and H - unsubstituted), charges (q = N2 for dianion, P2 for dication, 0 for neutral, N1 for anion, and P1 for cation), S or T refers to singlet or triplet state and b refers to B3LYP functional.

Experimental Procedure for Macrocyclization

Scheme S1

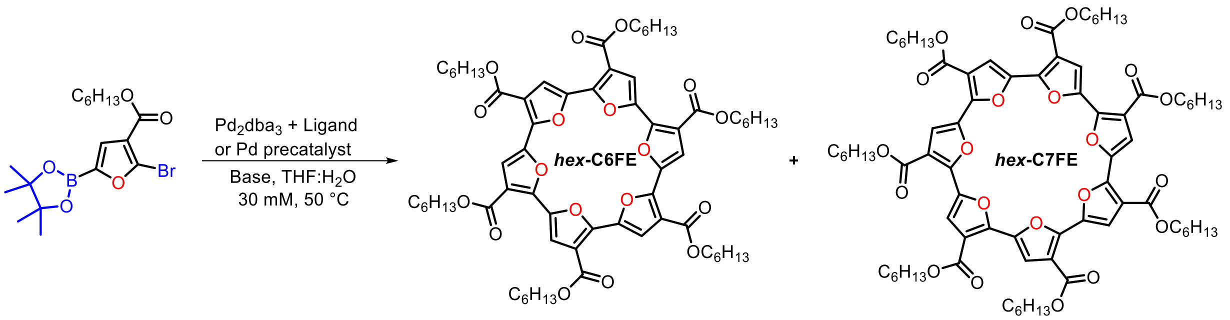

Representative Procedure corresponding to results reported in Table 1. In a nitrogen-filled glovebox, a 20 mL scintillation vial was charged with the monomer (50 mg, 0.125 mmol), trimethoxybenzene (4 mg, 0.024 mmol), and THF (2 mL). A 0.1 mL aliquot of this solution was diluted with 0.5 mL of CD_2Cl_2 for ^{1}H NMR spectroscopy to determine the precise ratio of the internal standard relative to the monomer (t = 0).

- *When using Pd_2dba_3:* Base (CsF or K_3PO_4, 0.374 mmol) and ligand ([(*t*-Bu)$_3$PH]BF_4, PCy_3, or PAd_3, 0.012 mmol) were added directly to the reaction mixture. Separately, Pd_2dba_3 (6 mg, 0.006 mmol) was crushed into a fine powder, dissolved in 1.5 mL of THF, and transferred to the mixture.
- *When not using Pd_2dba_3 (Table 1, Entries 1, 2, 3, and 11):* Base (CsF or K_3PO_4, 0.374 mmol), catalyst (G3PdSPhos, 0.01 mmol, or G3PdP(*t*-Bu)$_3$, 0.012 mmol), and 1.5 mL of THF (2 mL for Entry 1) were added directly to the reaction mixture.

With all the reagents added, the vial was sealed with a septum-sealed cap, removed from the glovebox. Finally, 0.65 mL of degassed (nitrogen-purged) deionized water was added. The mixture was then heated at 50 °C for 1 h in an oil bath. After the reaction, a 0.1 mL aliquot was diluted with 0.5 mL of CD_2Cl_2 to estimate the monomer-to-macrocycle conversion using ^{1}H NMR spectroscopy.

To determine conversion, the integration of the monomer peak in the t = 0 ^{1}H NMR spectrum was set to 100 (to denote 100% at the outset), and the integration of the internal standard was calculated relative to the monomer peak. The same integration value for the internal standard was used in the ^{1}H NMR spectrum recorded after reaction completion. The integrals for *hex*-C6FE peak ($\mathbf{I_6}$) and *hex*-C7FE peak ($\mathbf{I_7}$) were used to determine:

$$\textbf{Total Macrocycle Conversion (\%)} = \mathbf{I_6 + I_7}$$

For the entries in Table 1 with monomer-to-macrocycle conversions greater than 5%, reactions were repeated multiple times to confirm the reproducibility of the results. Representative crude ^{1}H NMR spectra for these high-conversion reactions, as well as those with conversions below 5%, are provided in Figures S6-15.

Detailed Procedure for Scale-up Synthesis. In a nitrogen-filled glovebox, a 100 mL Schlenk flask was charged with the monomer (250 mg, 0.623 mmol), trimethoxybenzene (21 mg, 0.125 mmol), and THF (5 mL). A 0.1 mL aliquot of this solution was diluted with 0.5 mL of CD_2Cl_2 for ^{1}H NMR spectroscopy to evaluate the internal standard relative to the monomer (t = 0). This step may be omitted if the monomer-to-macrocycle conversion is not required. CsF (284 mg, 1.87 mmol) and [(*t*-Bu)$_3$PH]BF_4 (ligand, 18 mg, 0.062 mmol) were then added to the same flask. Separately, a 20 mL scintillation vial was charged with $Pd_2(dba)_3$ (29 mg, 0.031 mmol) crushed into a fine powder. This was dissolved in 12.5 mL of THF and transferred to the 100 mL Schlenk flask. The Schlenk flask was sealed with a rubber septum, removed from the glovebox, and 3.5 mL of degassed, deionized water was added. The mixture was then heated at 50 °C for 1 h in an oil bath.

After the reaction, a 0.1 mL aliquot was diluted with 0.5 mL of CD_2Cl_2 to estimate the monomer-to-macrocycle conversion using ^{1}H NMR spectroscopy. The reaction mixture was concentrated

via rotary evaporation, and methanol was added to precipitate the macrocycle and polymer products. The crude product was isolated by vacuum filtration to remove small-molecule byproducts and purified using column chromatography (hexanes: CH_2Cl_2, 1:0 to 1:1).

The macrocycles, *hex*-C6FE, and *hex*-C7FE, were separated as red and dark orange powders, respectively, and washed with acetonitrile to remove open-chain oligomers. Further purification was accomplished using solvent diffusion recrystallization (CH_2Cl_2: methanol, 1:3). The final products were collected as a red solid, *hex*-C6FE (34 mg, 28%), and a dark orange solid, *hex*-C7FE (21 mg, 17%).

***hex*-C6FE:** ^{1}H NMR (500 MHz, $CDCl_3$) δ 7.46 (s, 6H), 4.17 (t, J = 7.2 Hz, 12H), 1.77 – 1.68 (m, 12H), 1.44 – 1.28 (m, 36H), 0.94 – 0.87 (m, 18H).

$^{13}C\{^1H\}$ NMR (126 MHz, $CDCl_3$) δ 161.9, 149.1, 144.2, 116.4, 116.3, 65.1, 31.7, 28.8, 25.7, 22.7, 14.2.

MS (MALDI-TOF) *m/z:* $[M]^+$ Calcd for $C_{66}H_{84}O_{18}$ 1164.5658; found, 1164.4.

***hex*-C7FE:** ^{1}H NMR (500 MHz, $CDCl_3$) δ 7.99 (s, 7H), 4.28 (t, J = 7.0 Hz, 14H), 1.81 – 1.73 (m, 14H), 1.49 – 1.38 (m, 14H), 1.38 – 1.29 (m, 28H), 0.95 – 0.87 (m, 21H).

$^{13}C\{^1H\}$ NMR (126 MHz, $CDCl_3$) δ 162.3, 147.9, 143.2, 118.4, 117.3, 65.5, 31.7, 28.8, 25.8, 22.8, 14.2.

MS (MALDI-TOF) *m/z:* $[M]^+$ Calcd for $C_{77}H_{98}O_{21}$ 1358.6601; found, 1358.5.

Alternative Methods for Small-Scale Isolation and Purification of Macrocycles: For small-scale synthesis, column chromatography using hexanes:CH_2Cl_2 can be used to efficiently isolate the macrocycle mixture from polymer byproducts. Subsequent separation of *hex*-C6FE and *hex*-C7FE can be accomplished with preparative TLC using toluene as the mobile phase.

For large amounts of crude product, column chromatography with a slow gradient of hexanes: CH_2Cl_2 is essential for effectively separating *hex*-C6FE and *hex*-C7FE. Minimal separation for the spots corresponding to *hex*-C6FE, and *hex*-C7FE is noted in TLC with hexanes:CH_2Cl_2 (3:2), which necessitates precise control during chromatography to effectively isolate the two macrocycles. This is best if preparative TLC is impractical.

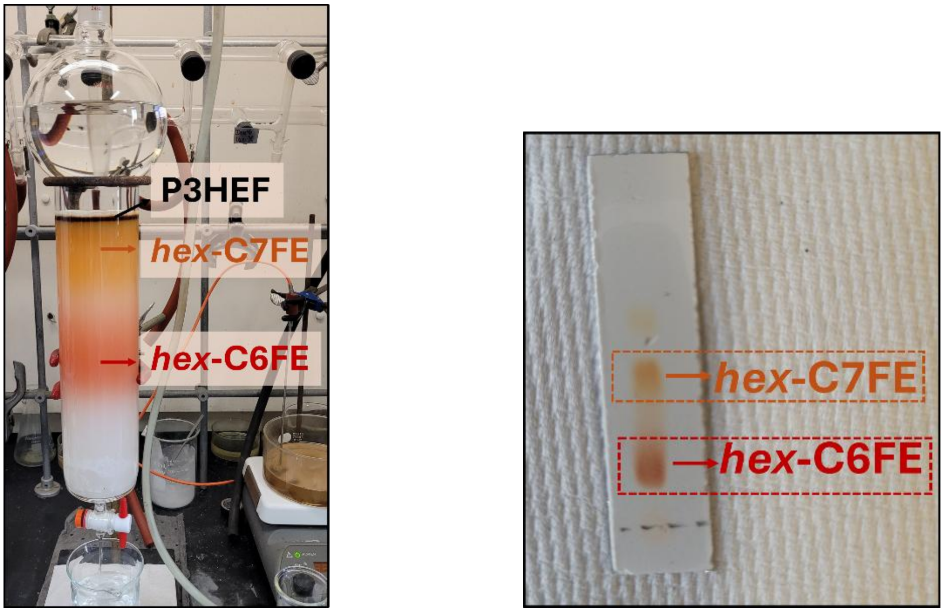

Figure S1. Left- Image of column chromatography to isolate and purify *hex*-C6FE and *hex*-C7FE using hexane: CD_2Cl_2 (1:1) as eluents. **Right-** Image of TLC plate showing separation of *hex*-C6FE and *hex*-C7FE using toluene as a mobile phase.

Experimental Procedure for Model Studies with ethyl-2,5-dibromofuran-3-carboxylate.

Scheme S2.

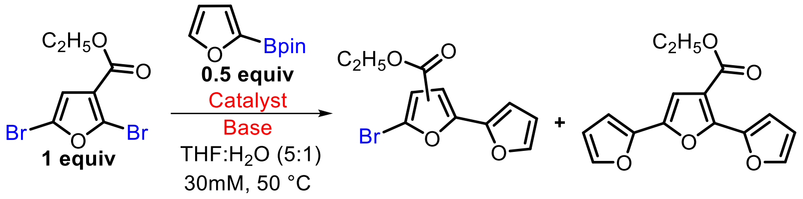

Representative Procedure. In a nitrogen-filled glovebox, a 20 mL scintillation vial was charged with ethyl-2,5-dibromofuran-3-carboxylate (50 mg, 0.168 mmol), furan-2-boronic acid pinacol ester (FuBpin, 16 mg, 0.082 mmol), trimethoxybenzene (internal standard, 12 mg, 0.071 mmol), and THF (3 mL). A 0.1 mL aliquot of this solution was diluted with diethyl ether and analyzed using GC-MS (t = 0). Base (0.503 mmol), catalyst, and THF (1.66 mL) were then added to the vial.

The vial was sealed with a septum cap, removed from the glovebox, and 0.93 mL of degassed (nitrogen-purged) deionized water was added. The mixture was immediately heated at 50 °C for 24 h. Subsequently, a 0.1 mL aliquot was removed and analyzed by GC-MS.

Note: For the monosubstituted bifuran product, two regioisomers are possible. Conversion was determined via GC-MS by comparing the FuBpin signal to the internal standard. Representative GC-MS chromatograms are shown in Figures S17-S21. The quantities of catalyst and base used are specified in Table S1.

Table S1. Quantities of catalysts and base used for model studies.

Entry	Catalyst	Base
**1**	G3PdSPhos (7 mol%, 0.012 mmol, 9 mg)	K_3PO_4 (0.504 mmol, 107 mg)
**2**	G3PdP*t*Bu_3 (7 mol%, 0.012 mmol, 7 mg)	K_3PO_4 (0.504 mmol, 107 mg)
**3**	Pd_2dba_3 (5 mol%, 0.008 mmol) + [(*t*-Bu)$_3$PH]BF_4 (0.016 mmol)	CsF (0.504 mmol, 77 mg)
**4**	Pd_2dba_3 (5 mol%, 0.008 mmol) + PAd_3 (0.016 mmol)	CsF (0.504 mmol, 77 mg)
**5**	Pd_2dba_3 (5 mol%, 0.008 mmol) + PCy_3 (0.016 mmol)	CsF (0.504 mmol, 77 mg)

NMR Spectra

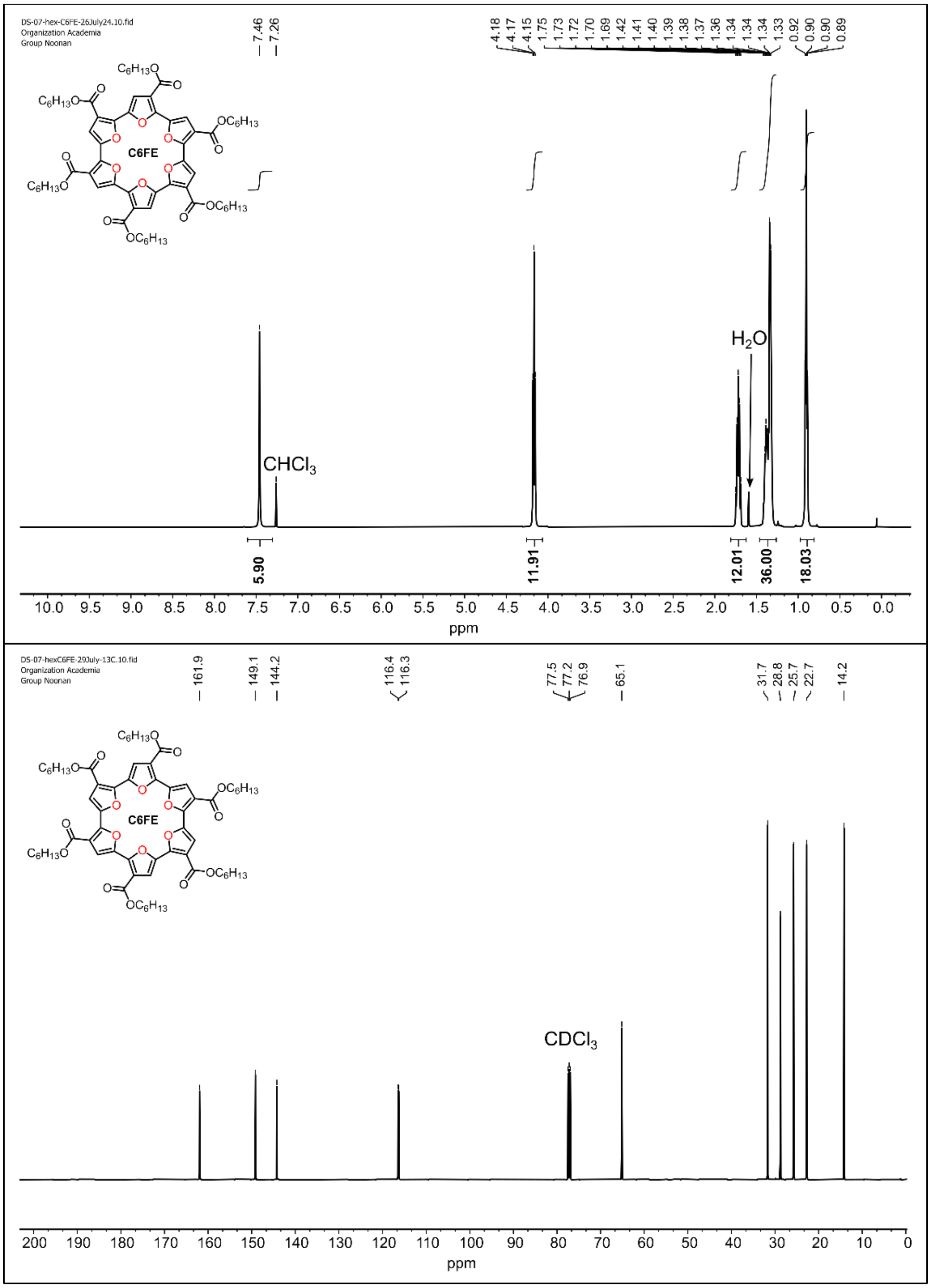

Figure S2. ^{1}H NMR spectrum (Top, 500 MHz, $CDCl_3$) and $^{13}C\{^{1}H\}$ NMR spectrum (Bottom, 126 MHz) of ***hex*-C6FE** collected at 300 K.

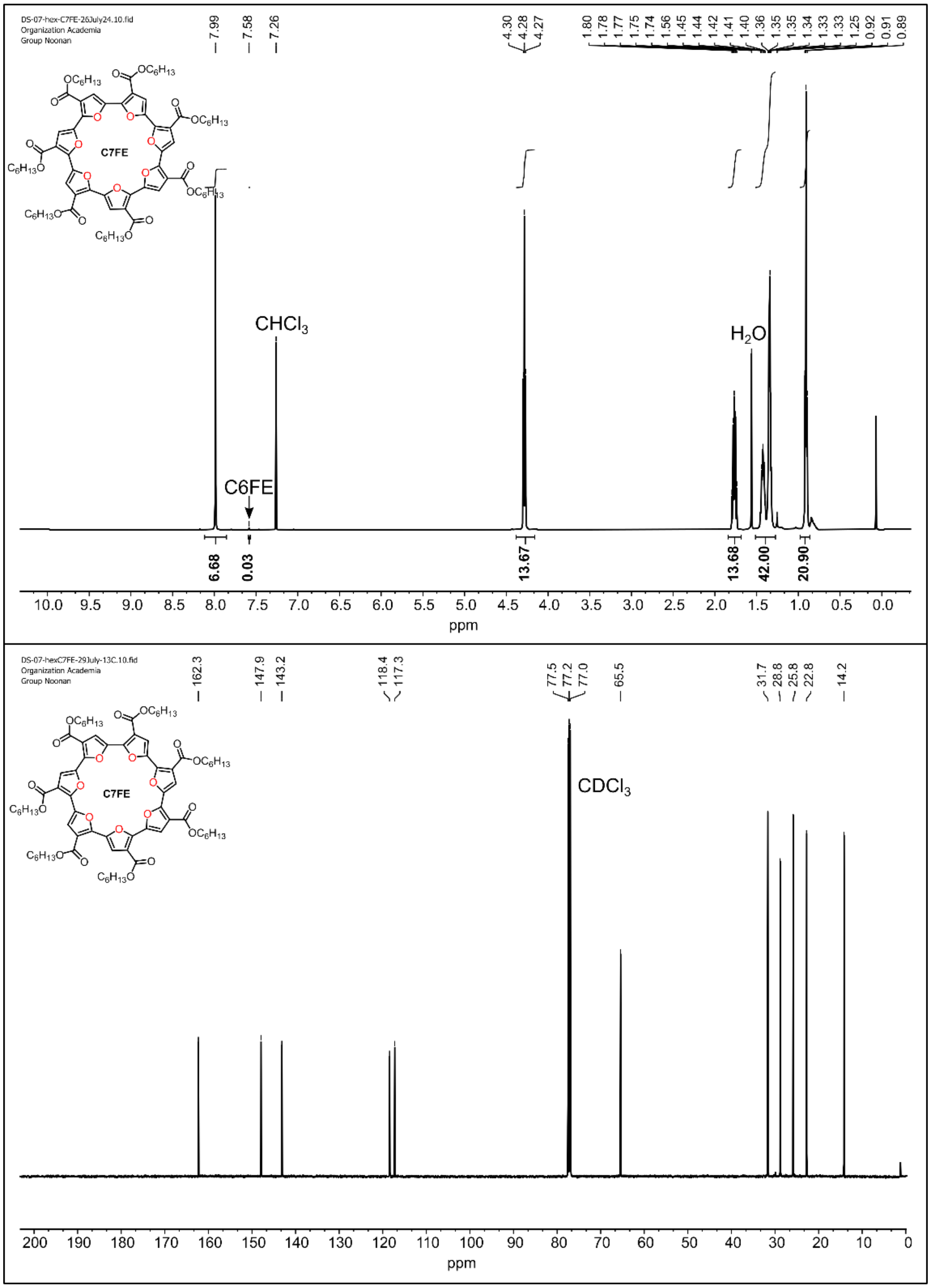

Figure S3. ^{1}H NMR spectrum (Top, 500 MHz, $CDCl_3$) and $^{13}C\{^{1}H\}$ NMR spectrum (Bottom, 126 MHz) of ***hex*-C7FE** collected at 300 K. A minor impurity (~0.5%) of *hex*-C6FE is marked as "C6FE" at 7.58 ppm in the ^{1}H NMR spectrum.

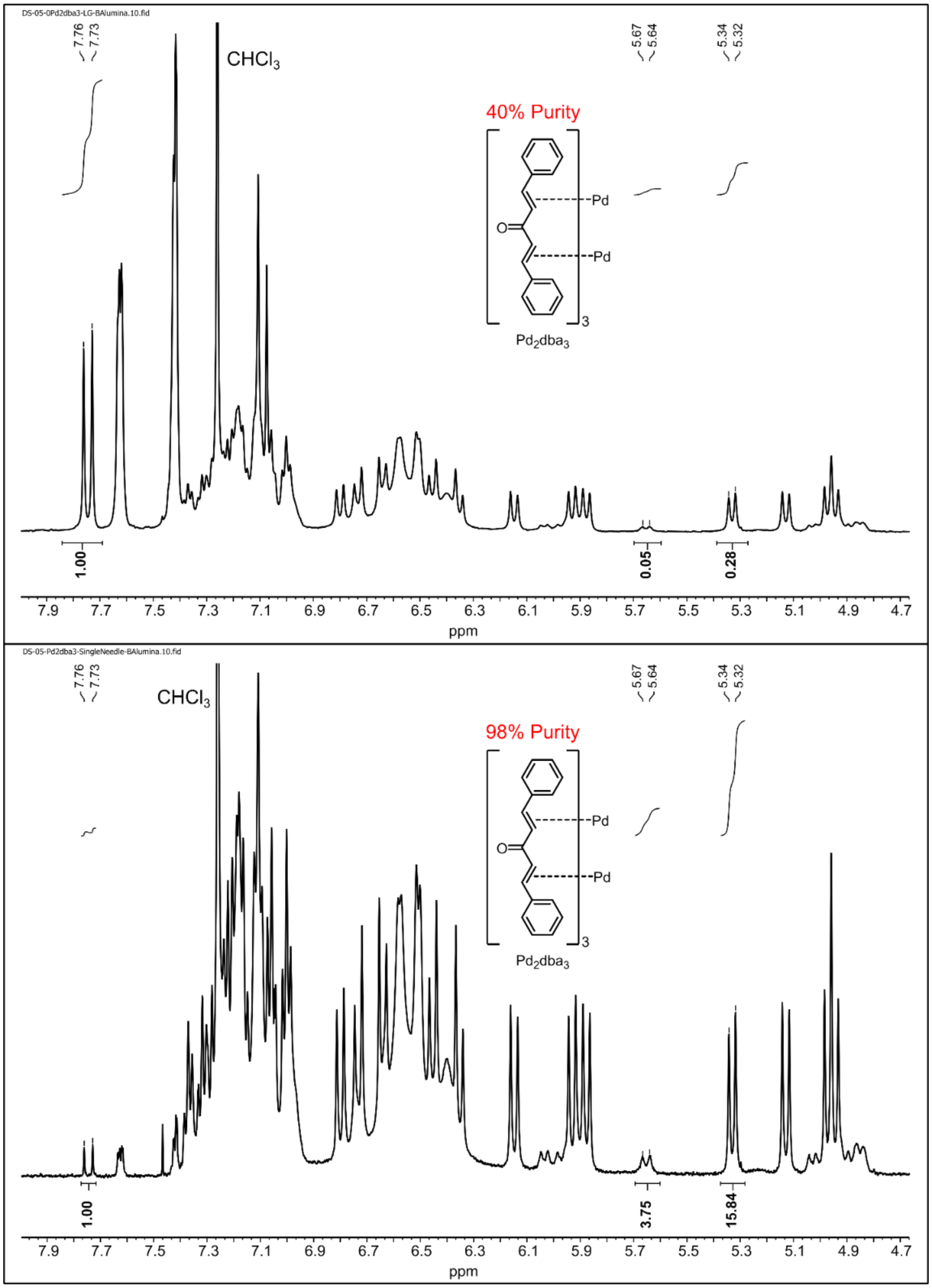

Figure S4. ^{1}H NMR spectrum (500 MHz, $CDCl_3$) of Pd_2dba_3 collected at 300 K. **Top:** commercial sample (40% purity). **Bottom:** recrystallized product (98% purity).

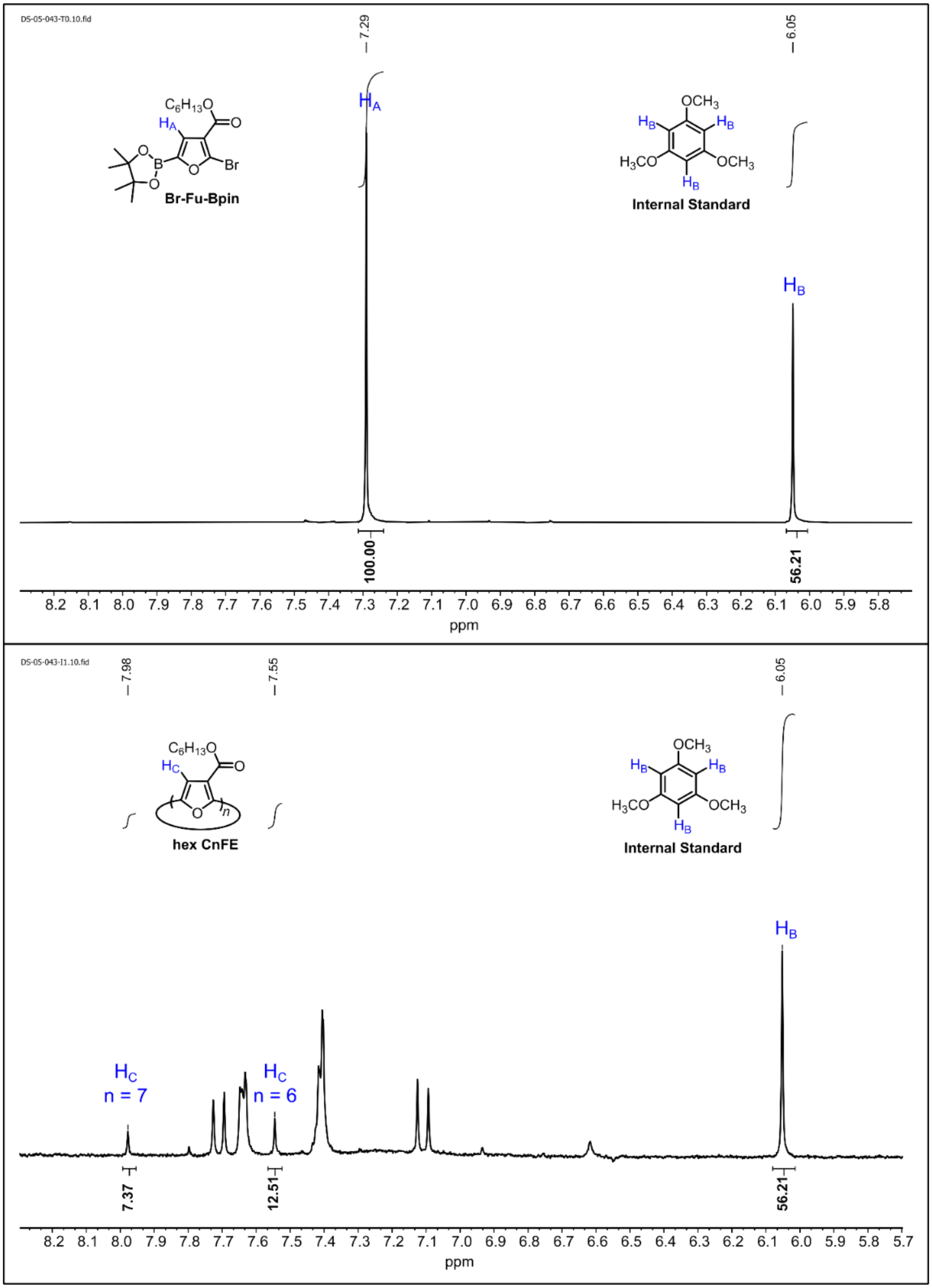

Figure S5. Crude ^{1}H NMR spectrum (500 MHz, CD_2Cl_2, 300 K) of the macrocyclization reaction using 40% pure Pd_2dba_3. **Top:** monomer to internal standard ratio at t = 0. **Bottom:** spectrum after reaction completion.

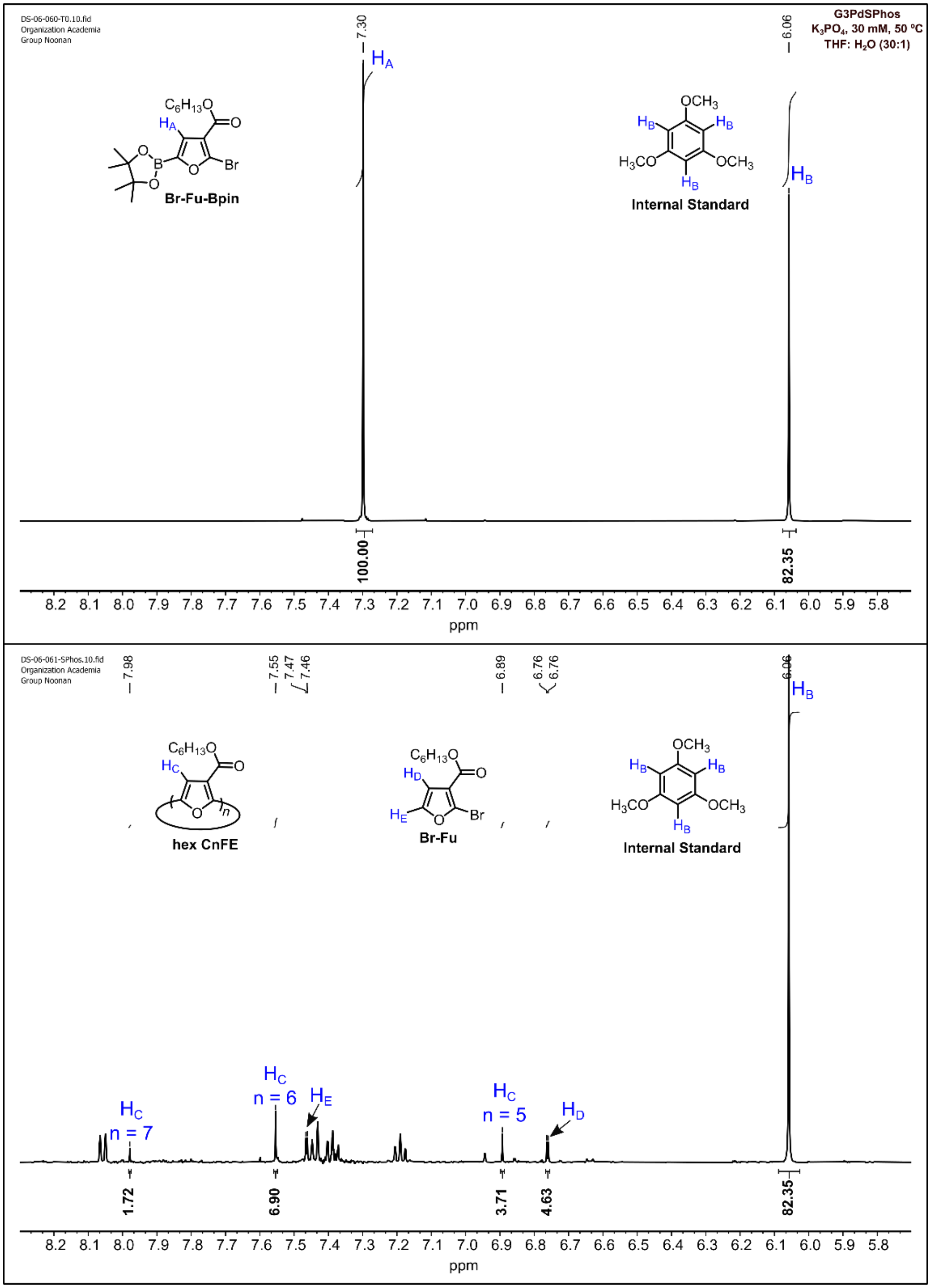

Figure S6. Representative crude ^{1}H NMR spectrum (500 MHz, CD_2Cl_2, 300 K) of the macrocyclization reaction using **G3PdSPhos** as the catalyst (Table 1, **Entry 1**). **Top:** monomer to internal standard ratio at t = 0. **Bottom:** spectrum after reaction completion.

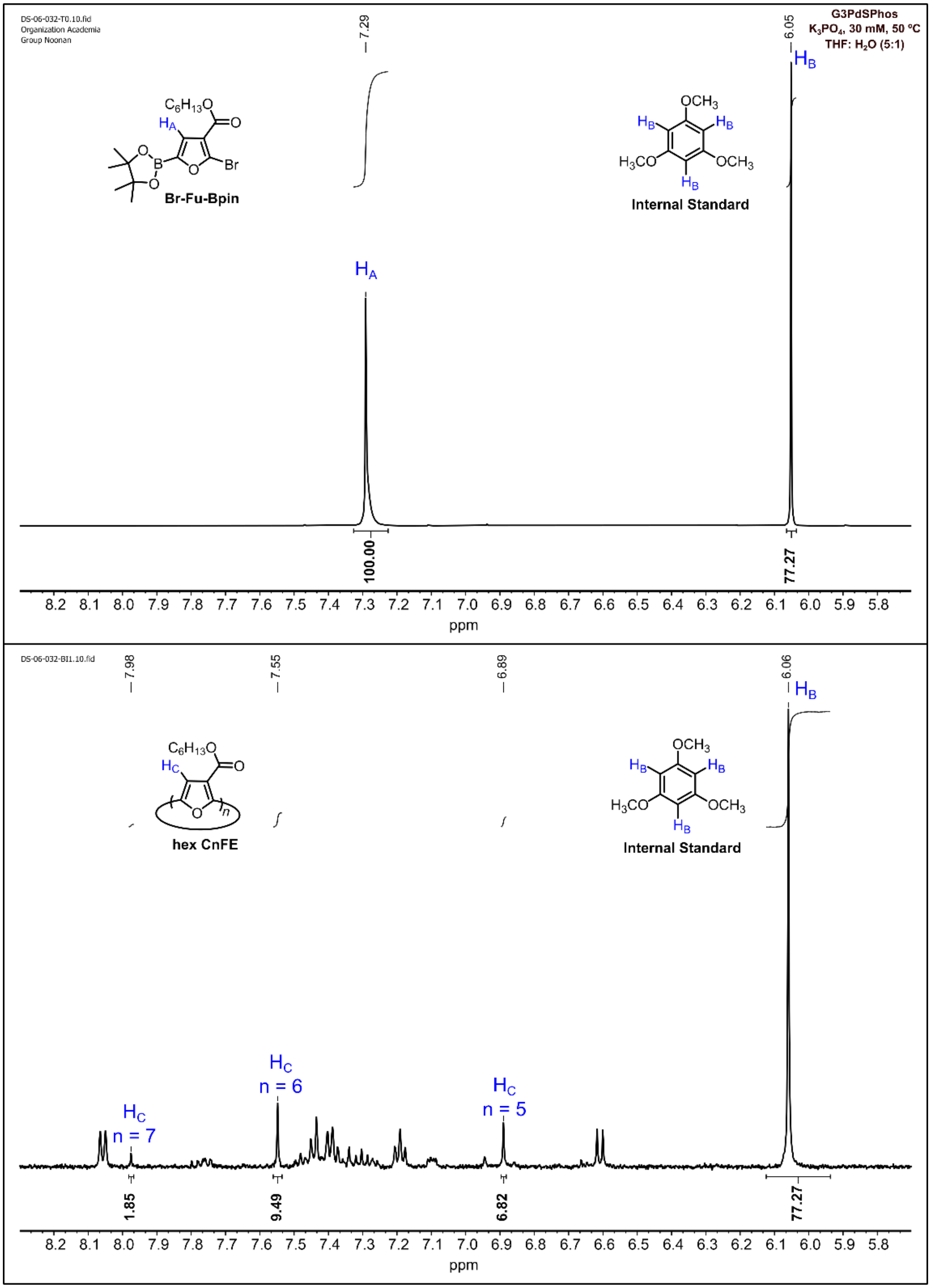

Figure S7. Representative crude ^{1}H NMR spectrum (500 MHz, CD_2Cl_2, 300 K) of the macrocyclization reaction using **G3PdSPhos** as the catalyst (Table 1, **Entry 2**). **Top:** monomer to internal standard ratio at t = 0. **Bottom:** spectrum after reaction completion.

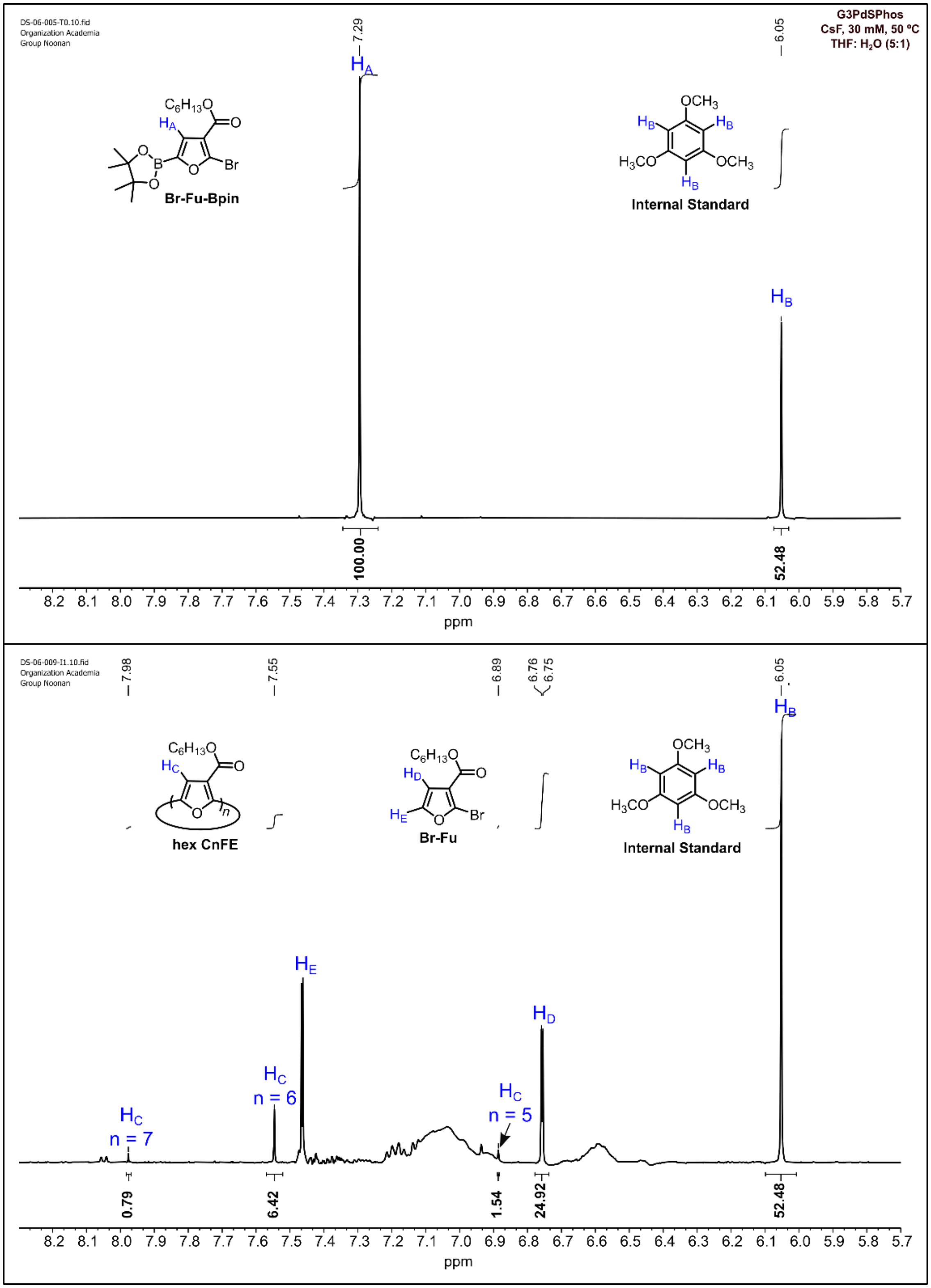

Figure S8. Representative crude ^{1}H NMR spectrum (500 MHz, CD_2Cl_2, 300 K) of the macrocyclization reaction using **G3PdSPhos** as the catalyst (Table 1, **Entry 3**). **Top:** monomer to internal standard ratio at t = 0. **Bottom:** spectrum after reaction completion.

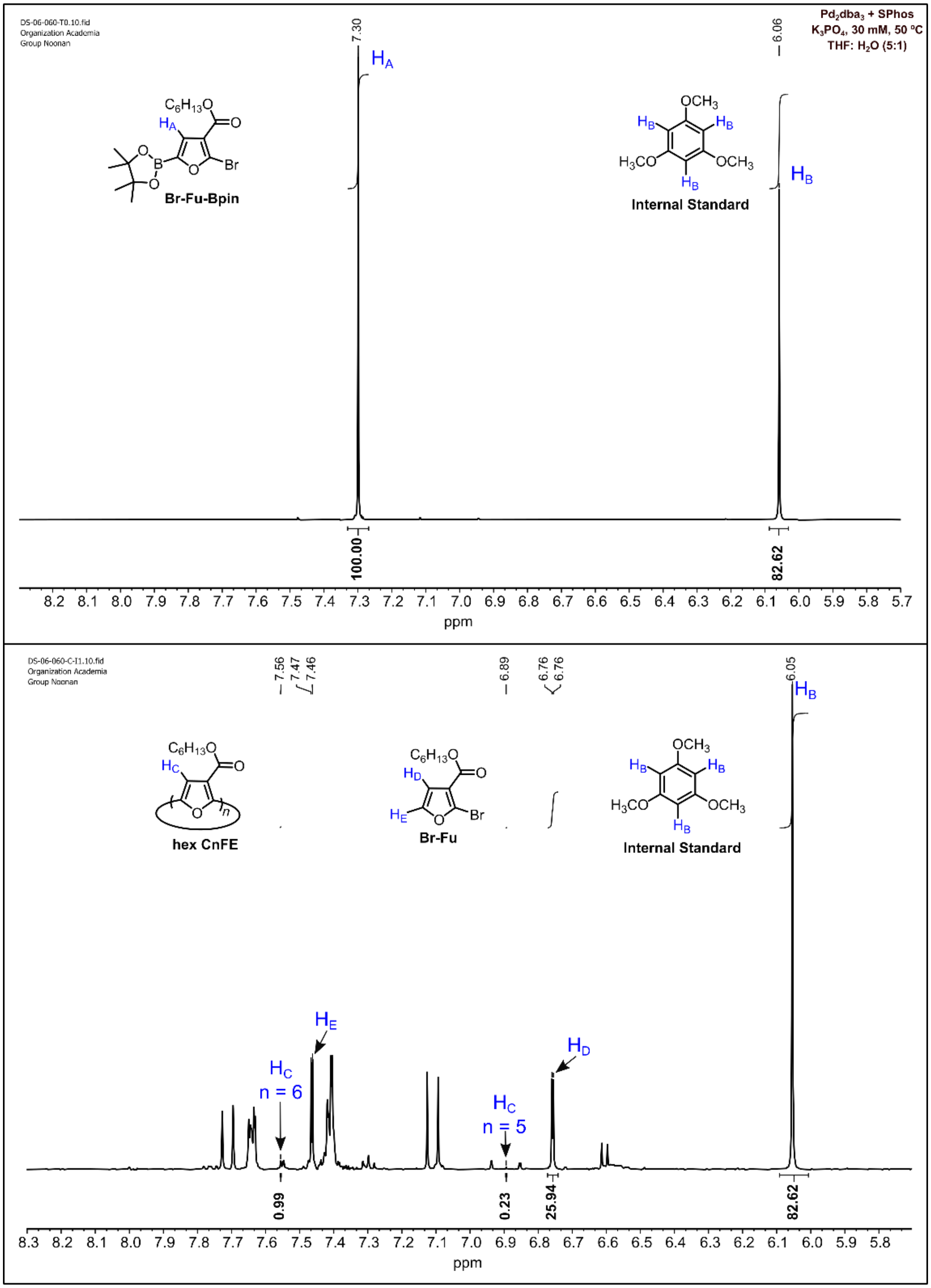

Figure S9. Representative crude ^{1}H NMR spectrum (500 MHz, CD_2Cl_2, 300 K) of the macrocyclization reaction using **Pd_2dba_3 + SPhos** as the catalyst (Table 1, **Entry 4**). **Top:** monomer to internal standard ratio at t = 0. **Bottom:** spectrum after reaction completion.

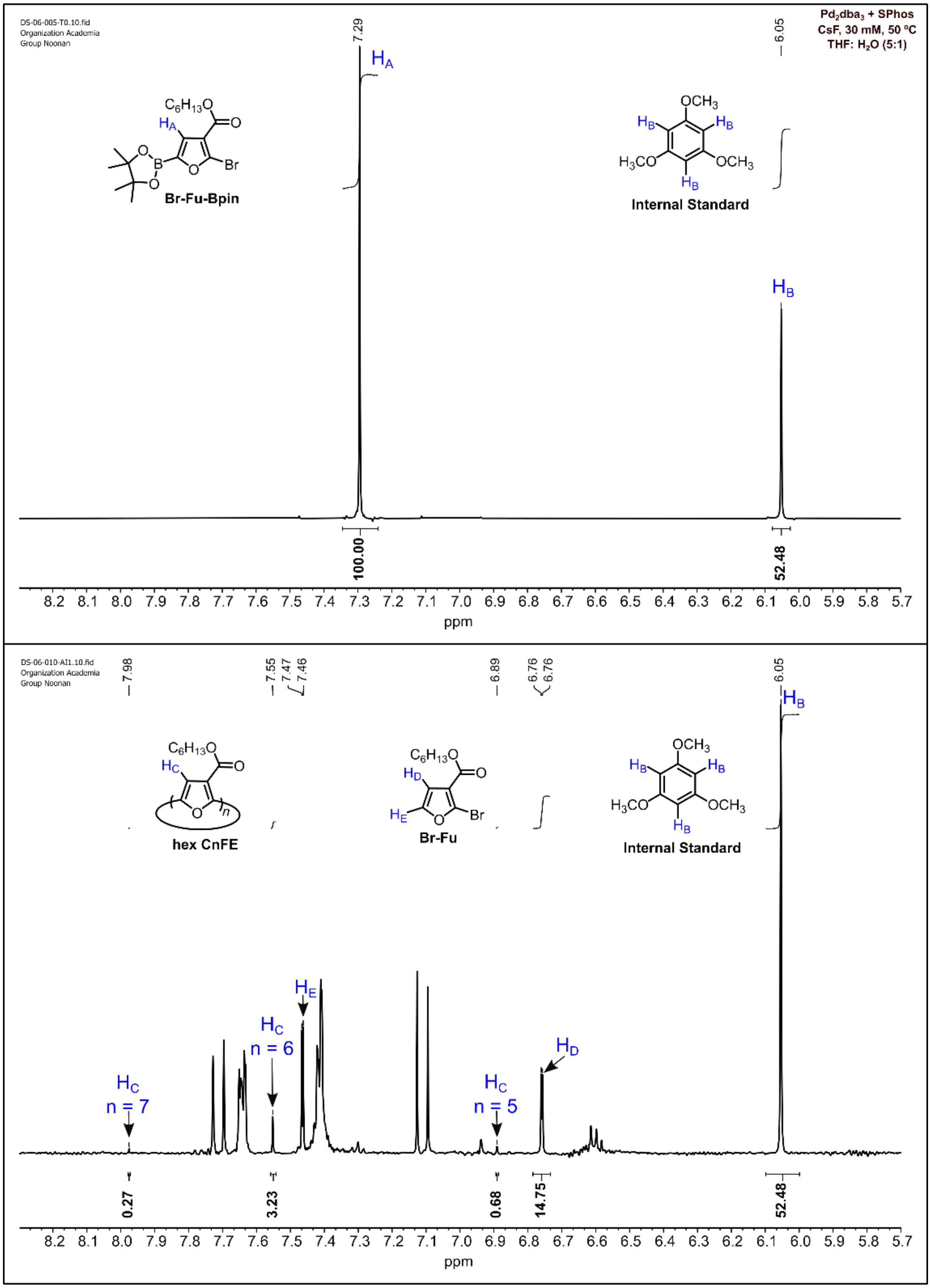

Figure S10. Crude ^{1}H NMR spectrum (500 MHz, CD_2Cl_2, 300 K) of the macrocyclization reaction using **Pd_2dba_3 + SPhos** as the catalyst (Table 1, **Entry 5**). **Top:** monomer to internal standard ratio at t = 0. **Bottom:** spectrum after reaction completion.

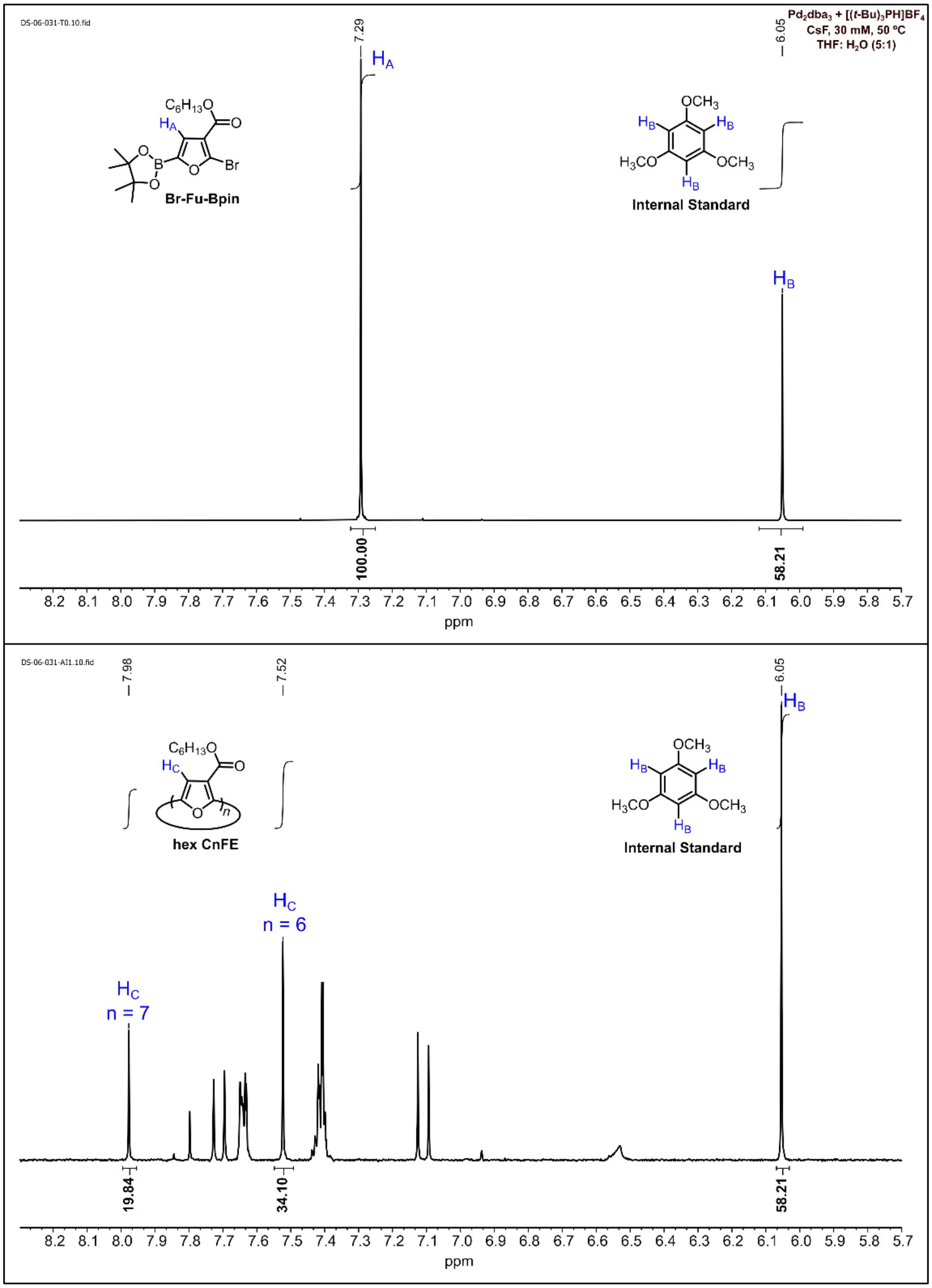

Figure S11. Representative crude ^{1}H NMR spectrum (500 MHz, CD_2Cl_2, 300 K) of the macrocyclization reaction using **Pd_2dba_3 + [(*t*-Bu)$_3$PH]BF$_4$** as the catalyst (Table 1, **Entry 6**). **Top:** monomer to internal standard ratio at t = 0. **Bottom:** spectrum after reaction completion.

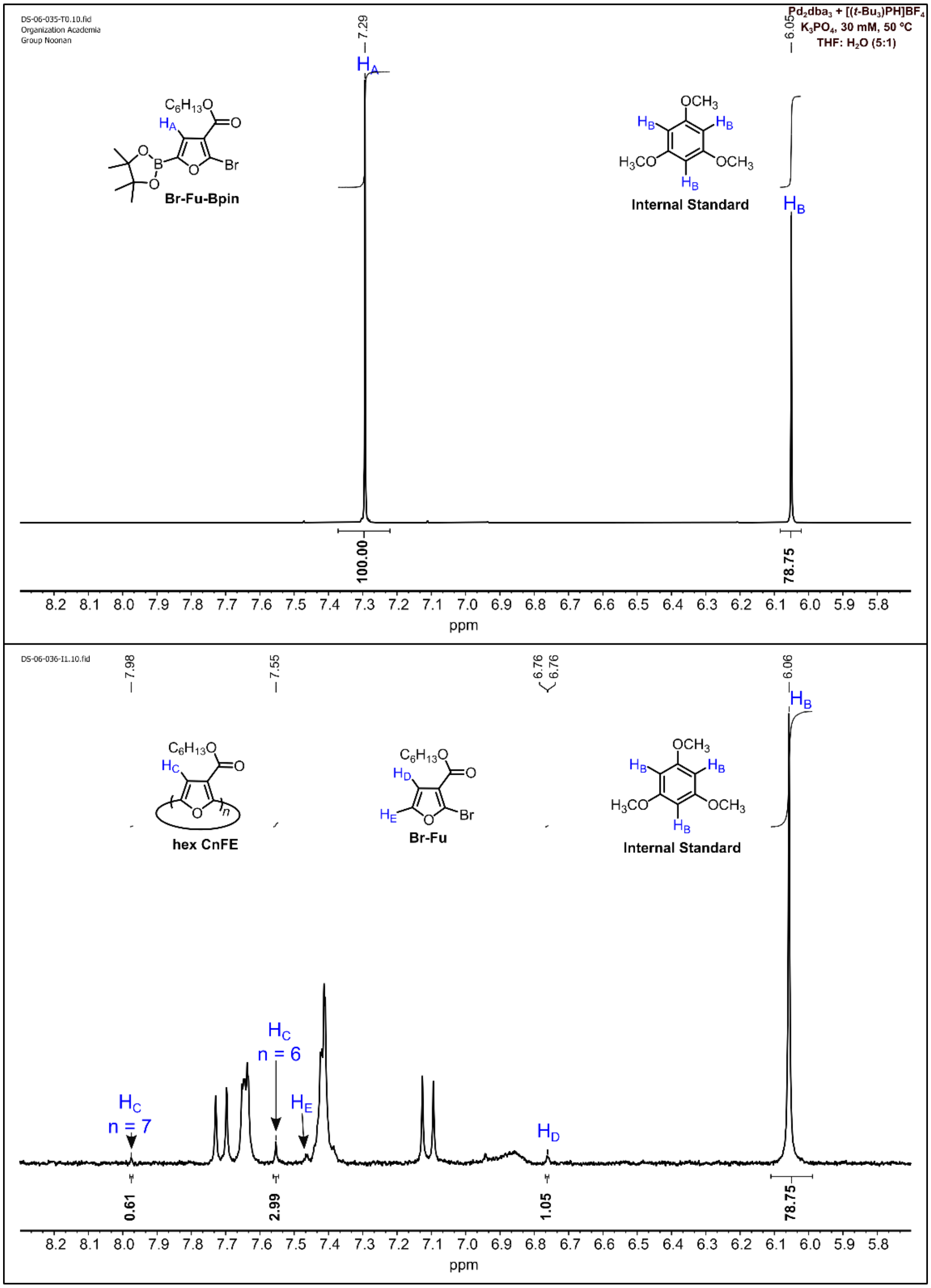

Figure S12. Crude ^{1}H NMR spectrum (500 MHz, CD_2Cl_2, 300 K) of the macrocyclization reaction using **Pd_2dba_3 + [(*t*-Bu)$_3$PH]BF_4** as the catalyst (Table 1, **Entry 7**). **Top:** monomer to internal standard ratio at t = 0. **Bottom:** spectrum after reaction completion.

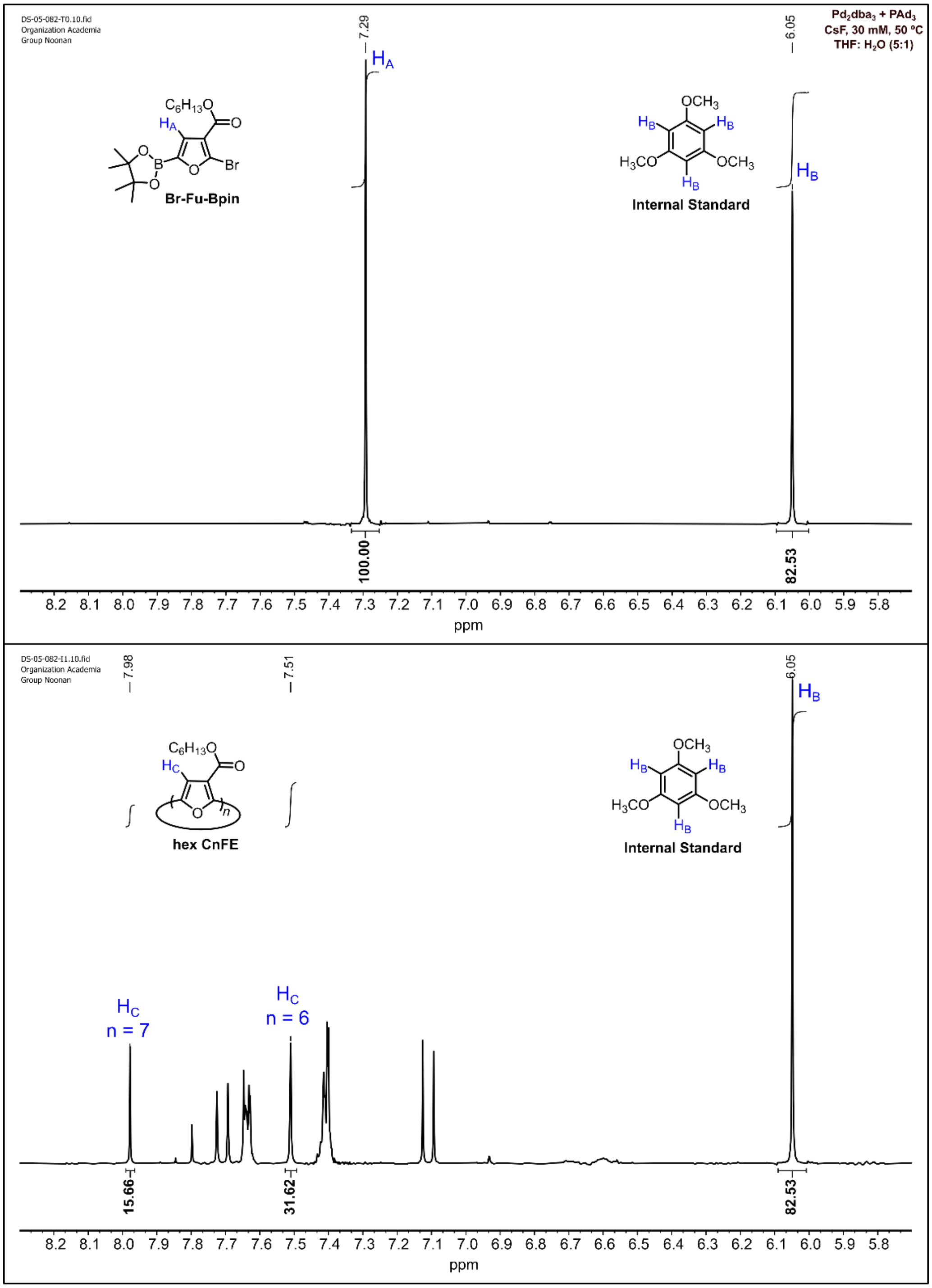

Figure S13. Representative crude ^{1}H NMR spectrum (500 MHz, CD_2Cl_2, 300 K) of the macrocyclization reaction using **Pd_2dba_3 + PAd_3** as the catalyst (Table 1, **Entry 8**). **Top:** spectrum at t = 0, before the reaction starts. **Bottom:** spectrum after the reaction completion.

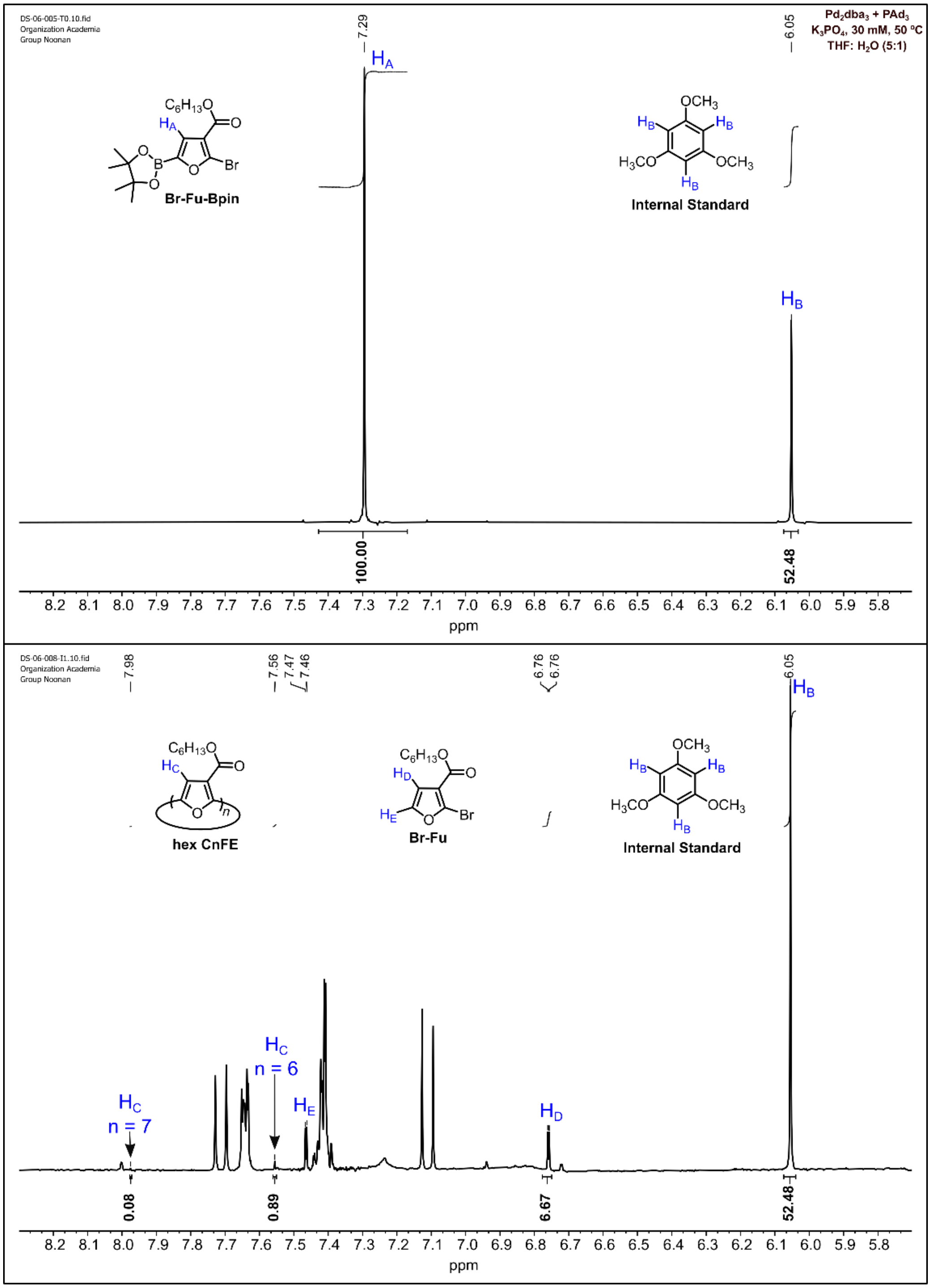

Figure S14. Crude ^{1}H NMR spectrum (500 MHz, CD_2Cl_2, 300 K) of the macrocyclization reaction using **Pd_2dba_3 + PAd_3** as the catalyst (Table 1, **Entry 9**). **Top:** spectrum at t = 0, before the reaction starts. **Bottom:** spectrum after the reaction completion.

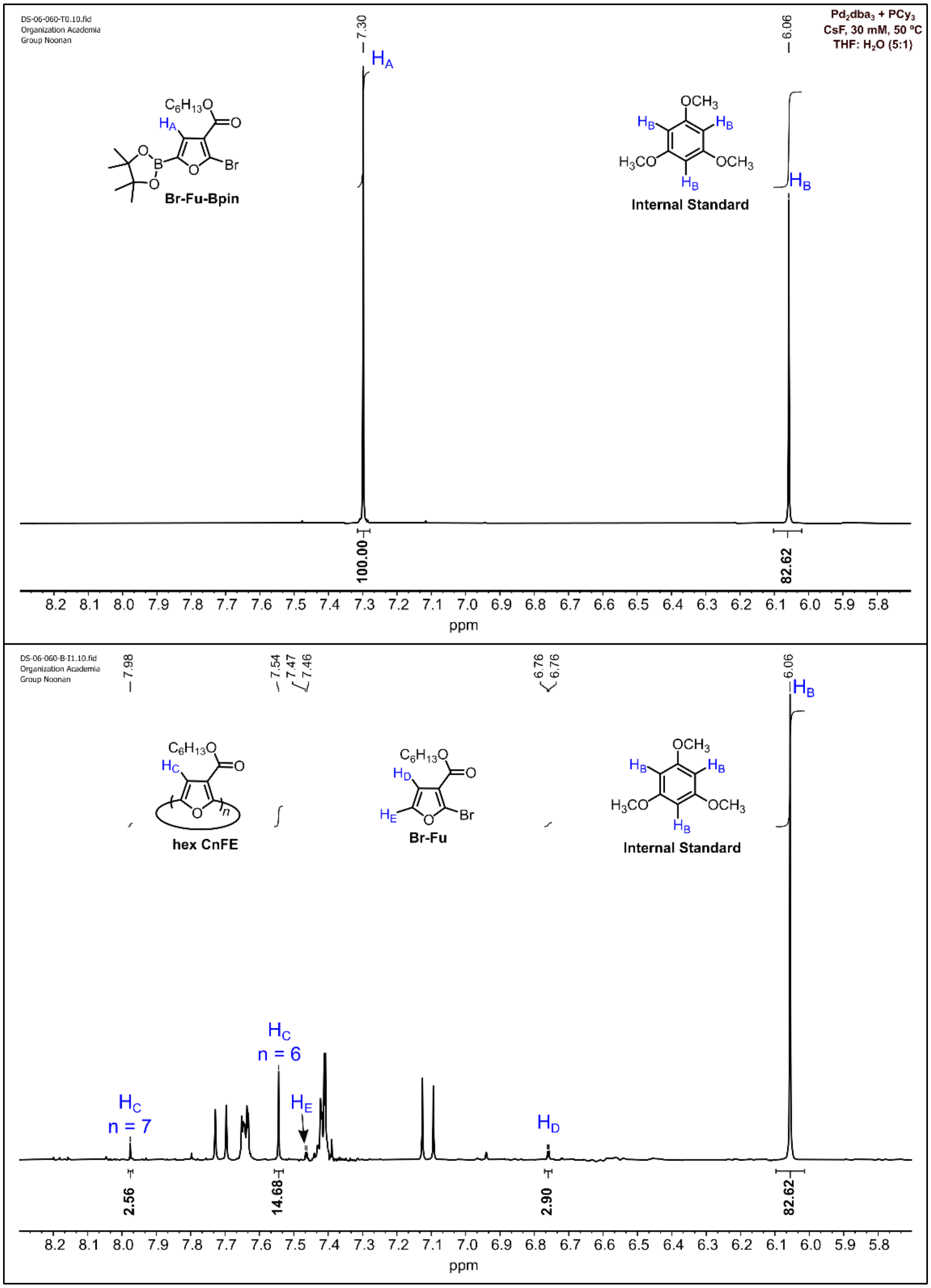

Figure S15. Representative crude ^{1}H NMR spectrum (500 MHz, CD_2Cl_2, 300 K) of the macrocyclization reaction using **Pd_2dba_3 + PCy_3** as the catalyst (Table 1, **Entry 10**). **Top:** spectrum at t = 0, before the reaction starts. **Bottom:** spectrum after the reaction completion.

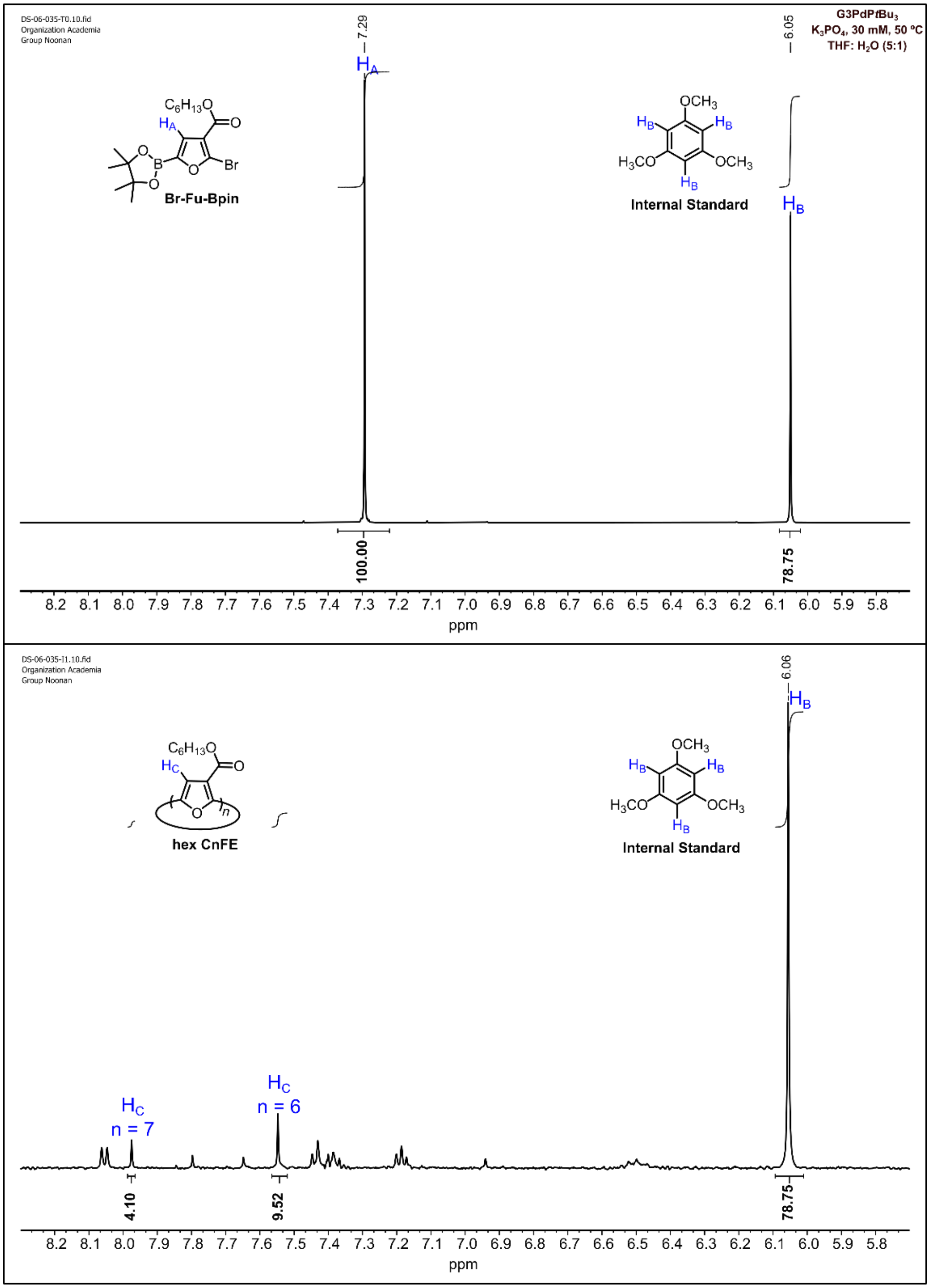

Figure S16. Representative crude ^{1}H NMR spectrum (500 MHz, CD_2Cl_2 , 300 K) of the macrocyclization reaction using **G3PdP(*t*-Bu)3** as the catalyst (Table 1, **Entry 11**). **Top:** monomer to internal standard ratio at t = 0. **Bottom:** spectrum after reaction completion.

GC-MS Chromatograms

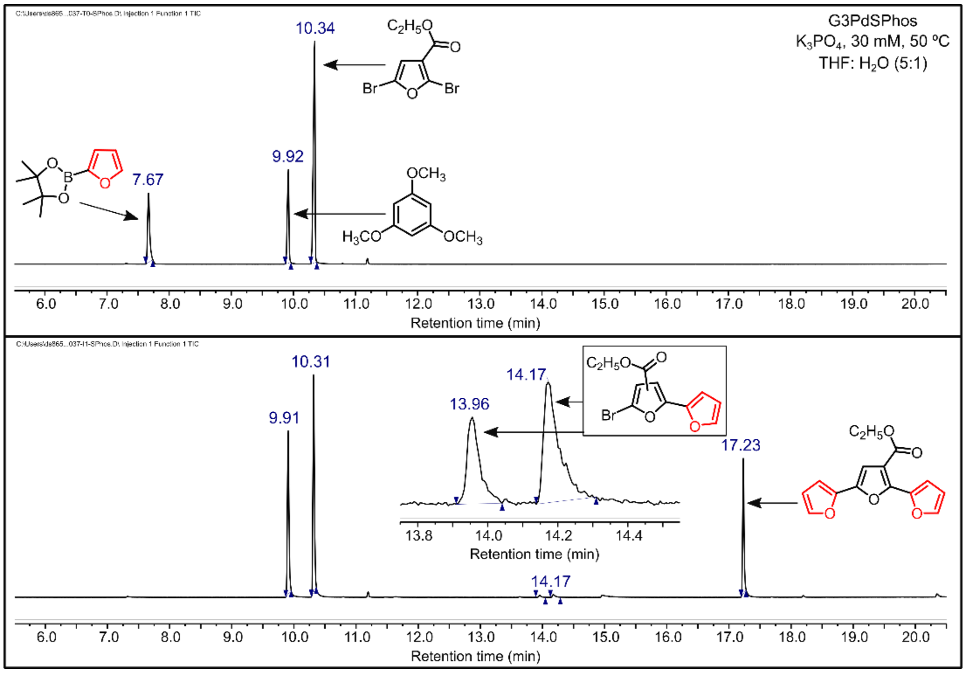

Figure S17. GC-MS chromatograms for model compound Suzuki-Miyaura coupling at 50 °C using ethyl-2,5-dibromofuran-3-carboxylate and G3PdSPhos (Table S2, **Entry 1).** Top – reaction mixture at time = 0 h. Bottom – reaction mixture after 24 h.

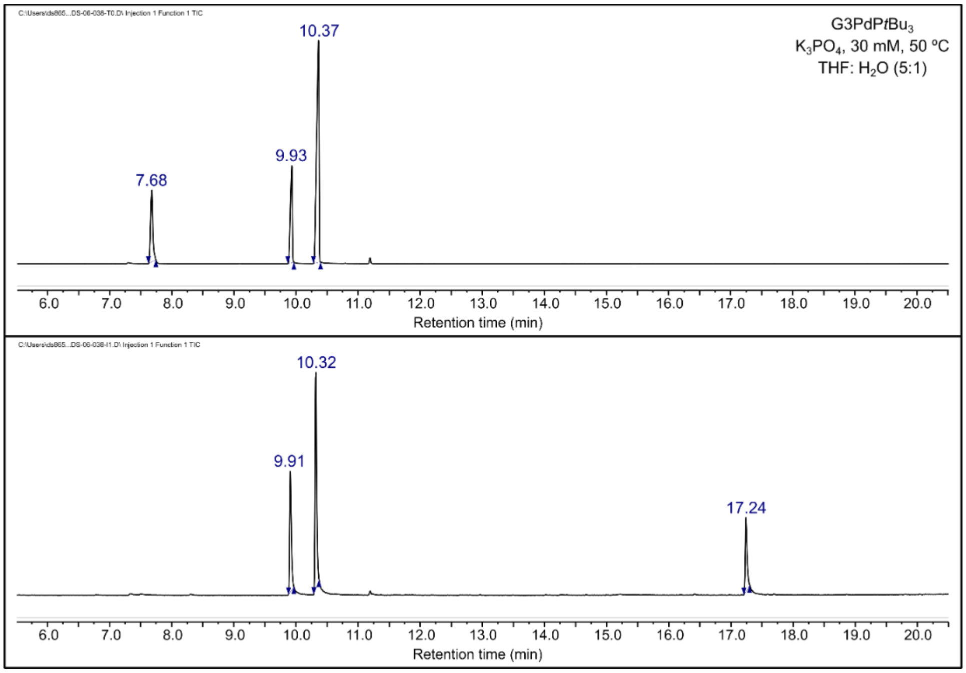

Figure S18. GC-MS chromatograms for model compound Suzuki-Miyaura coupling at 50 °C using ethyl-2,5-dibromofuran-3-carboxylate and G3PdP(t-Bu)$_3$ (Table S2, **Entry 2).** Top – reaction mixture at time = 0 h. Bottom – reaction mixture after 24 h.

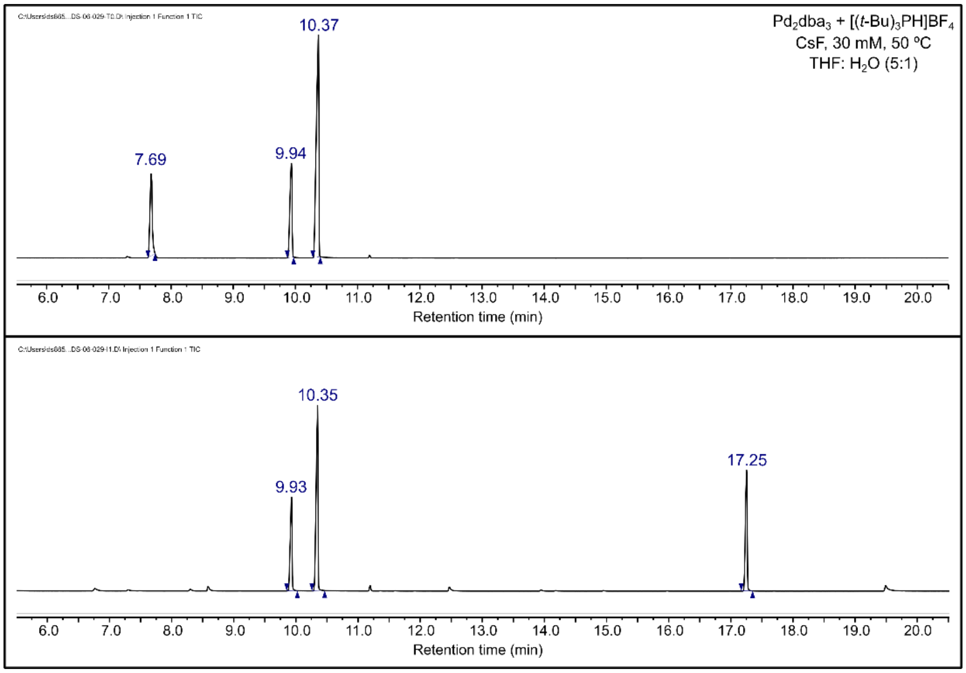

Figure S19. GC-MS chromatograms for model compound Suzuki-Miyaura coupling at 50 °C using ethyl-2,5-dibromofuran-3-carboxylate and Pd_2dba_3 + $[(t\text{-}Bu)_3PH]BF_4$ (Table S2, **Entry 3).** Top – reaction mixture at time = 0 h. Bottom – reaction mixture after 24 h.

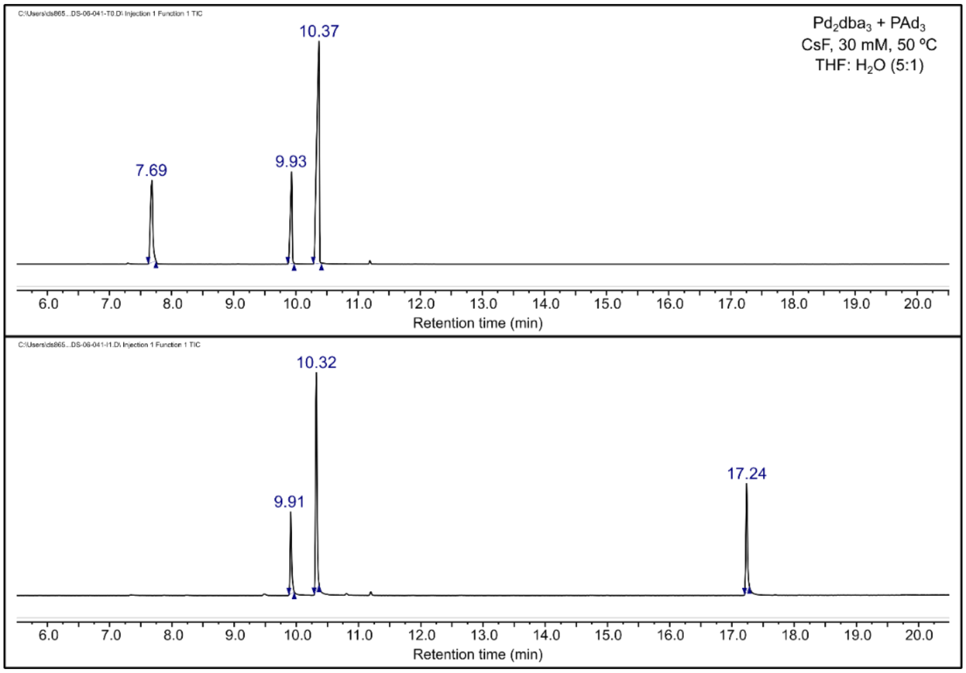

Figure S20. GC-MS chromatograms for model compound Suzuki-Miyaura coupling at 50 °C using ethyl-2,5-dibromofuran-3-carboxylate and Pd_2dba_3 + PAd_3 (Table S2, **Entry 4).** Top – reaction mixture at time = 0 h. Bottom – reaction mixture after 24 h.

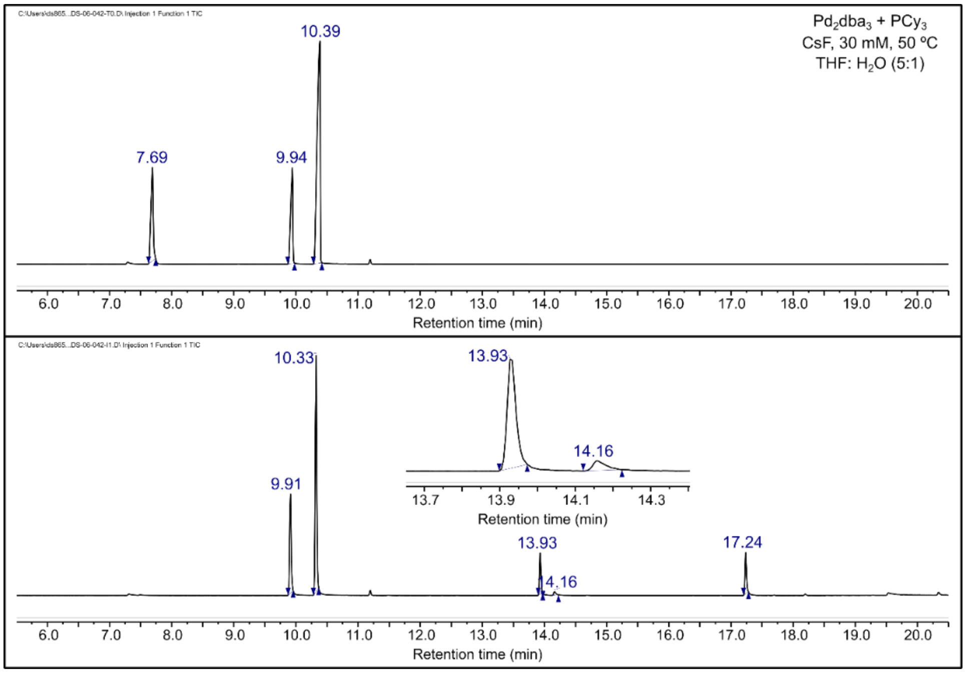

Figure S21. GC-MS chromatograms for model compound Suzuki-Miyaura coupling at 50 °C using ethyl-2,5-dibromofuran-3-carboxylate and Pd_2dba_3 + PCy_3 (Table S2, **Entry 5).** Top – reaction mixture at time = 0 h. Bottom – reaction mixture after 24 h.

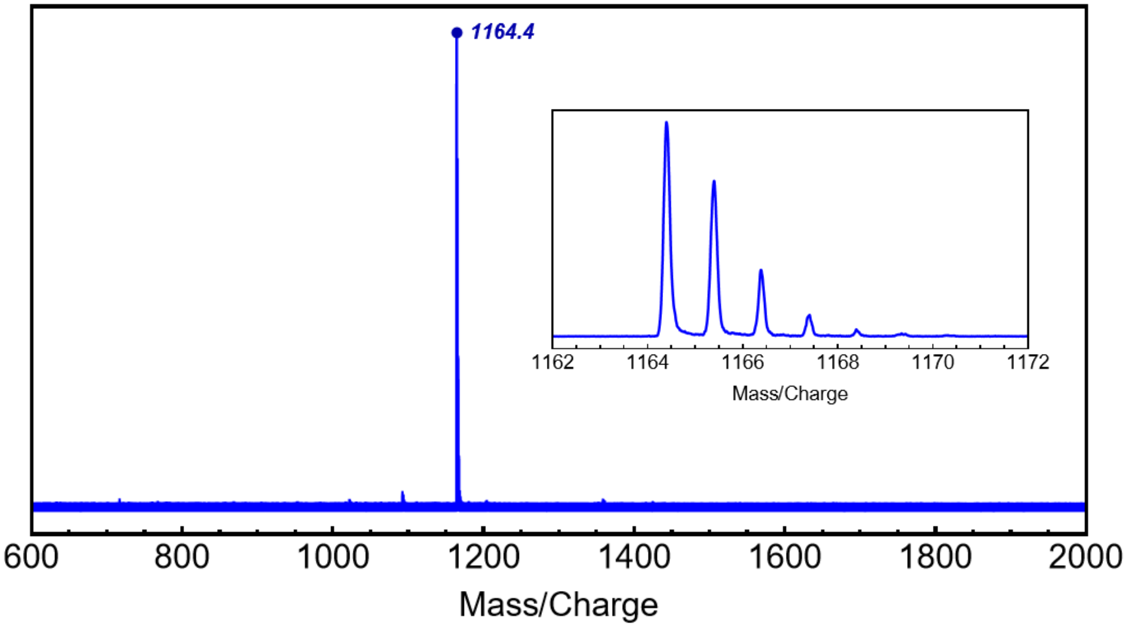

Figure S22. MALDI-TOF mass spectrum of *hex*-C6FE.

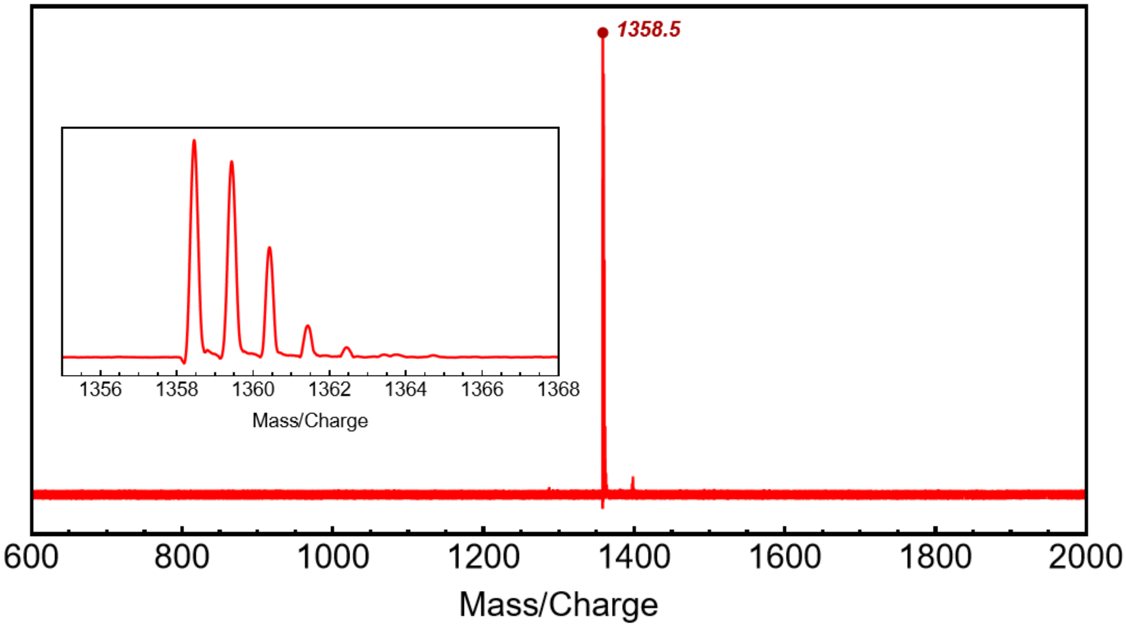

Figure S23. MALDI-TOF mass spectrum of *hex*-C7FE.

Table S2. Model Compound Reactions with ethyl-2,5-dibromofuran-3-carboxylate.

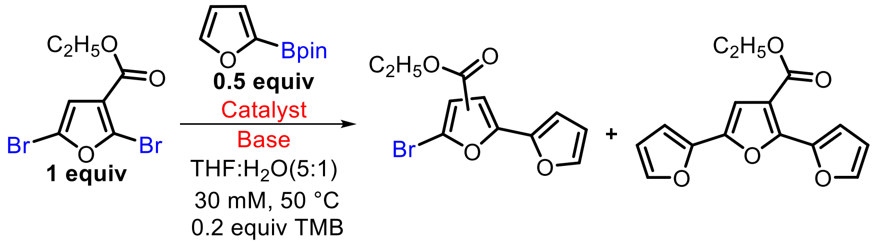

Entry	Catalyst[a] (mol %)	Base	percent conversion (%)	percent terfuran (%)
1	G3PdSPhos (7)	K_3PO_4	100	94
2	G3PdP(*t*-Bu)$_3$ (7)	K_3PO_4	100	99
3	Pd_2dba_3 (5) + [(*t*-Bu)$_3$PH]BF_4 (10)	CsF	100	98
4	Pd_2dba_3 (5) + PAd_3 (10)	CsF	100	>99
5	Pd_2dba_3 (5) + PCy_3 (10)	CsF	100	47

Conversion was determined by GC-MS using TMB as the internal standard. [a]Catalyst loading relative to ethyl-2,5-dibromofuran-3-carboxylate.

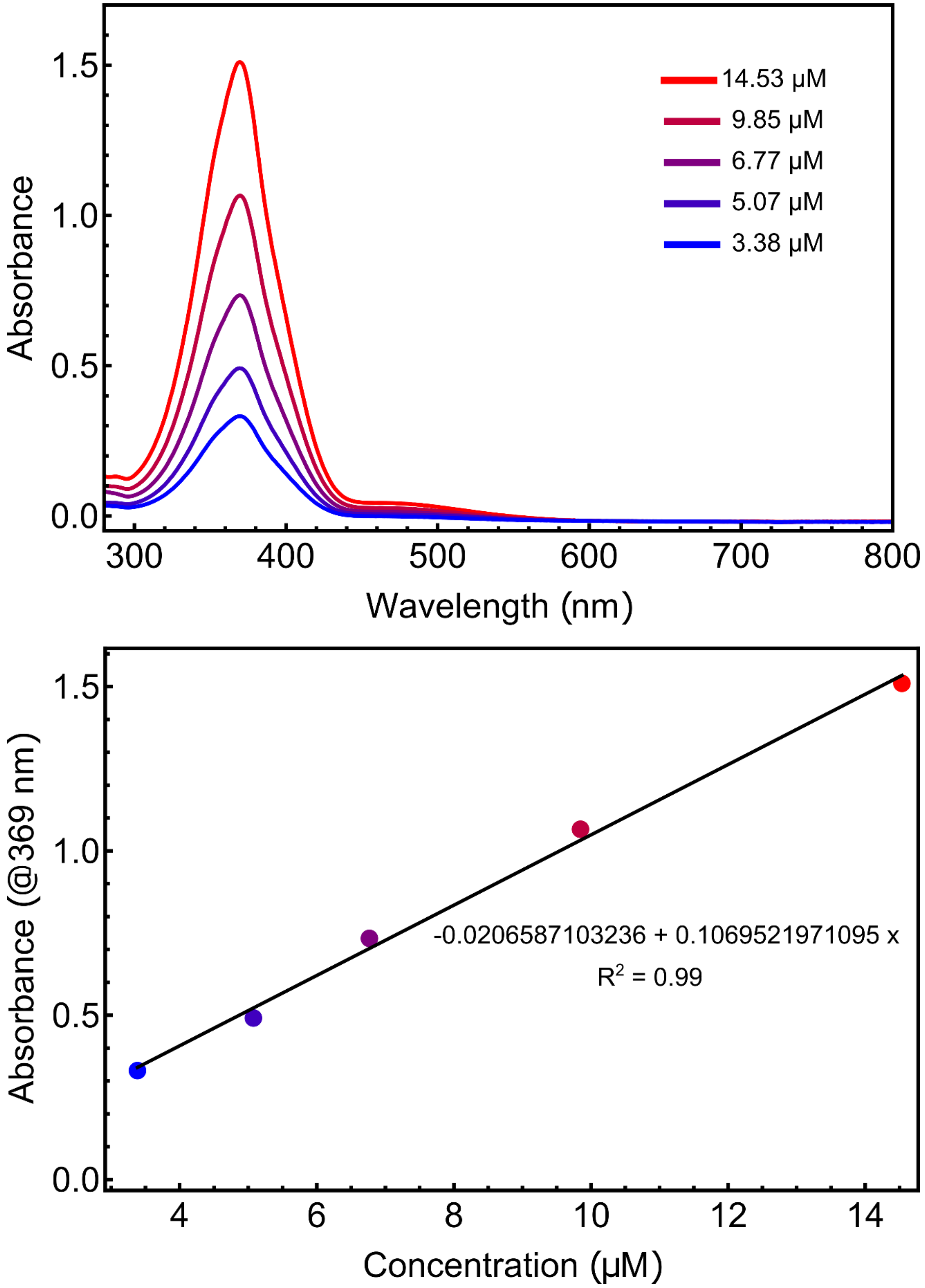

Figure S24. Top: Absorbance spectrum of *hex*-C7FE at varying concentrations in $CHCl_3$. **Bottom:** Plot of Abs. versus Conc. for *hex*-C7FE.

Cyclic Voltammetry

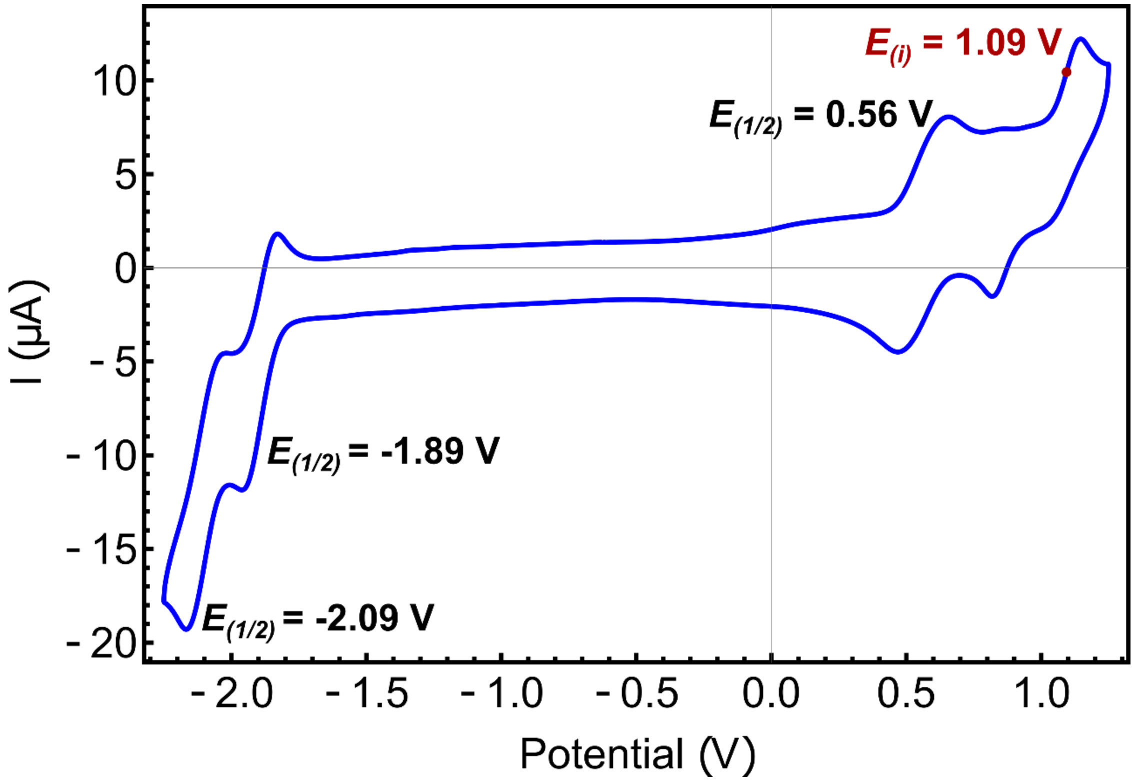

Figure S25. Cyclic voltammogram of *hex*-C6FE in degassed CH_2Cl_2 (0.63 mg/mL) using NBu_4PF_6 as the supporting electrolyte (0.07M), with a scan rate of 100 mV/s. The voltammogram was referenced using Fc/Fc^+ as an internal standard. IUPAC plotting convention, A 1 mm^2 glassy carbon working electrode, a platinum coil counter electrode, and a silver wire pseudo-reference electrode were employed for the measurements. The initial potential was ~ –0.62 V and swept to positive potentials (oxidative).

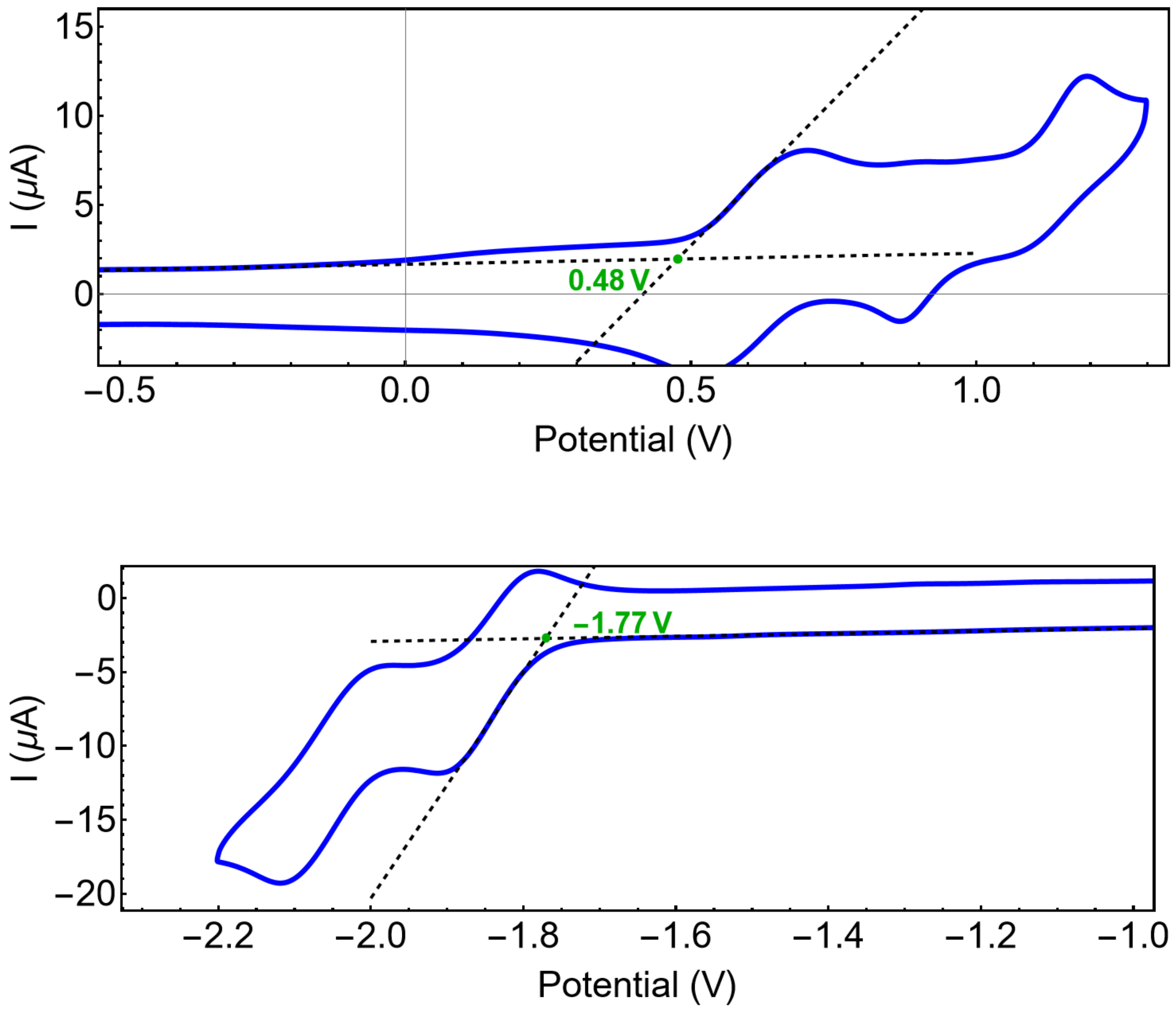

Figure S26. Determination of onset potentials for *hex*-C6FE from the CV shown in Figure S25.

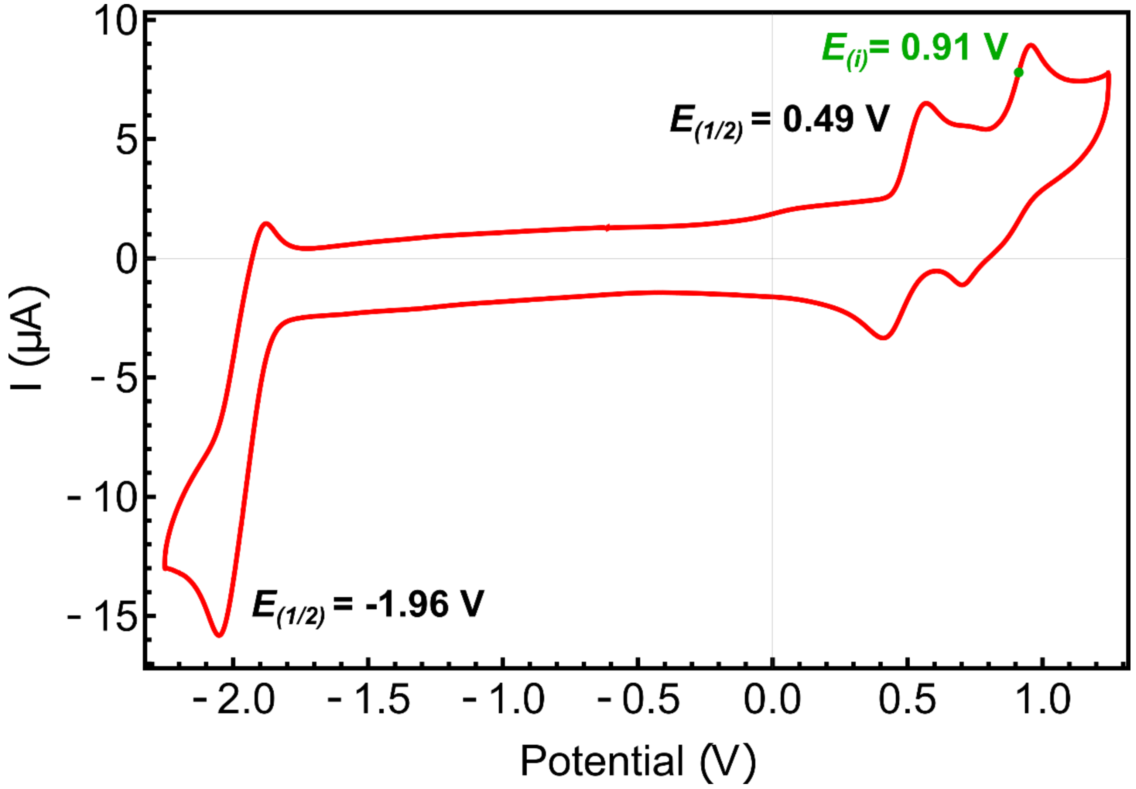

Figure S27. Cyclic voltammogram of *hex*-C7FE in degassed CH_2Cl_2 (0.63 mg/mL) using NBu_4PF_6 as the supporting electrolyte (0.07M), with a scan rate of 100 mV/s. The voltammogram was referenced using Fc/Fc^+ as an internal standard. A 1 mm^2 glassy carbon working electrode, a platinum coil counter electrode, and a silver wire pseudo-reference electrode were employed for the measurements. The initial potential was −0.62 V and scans were initially taken to positive potentials (oxidative).

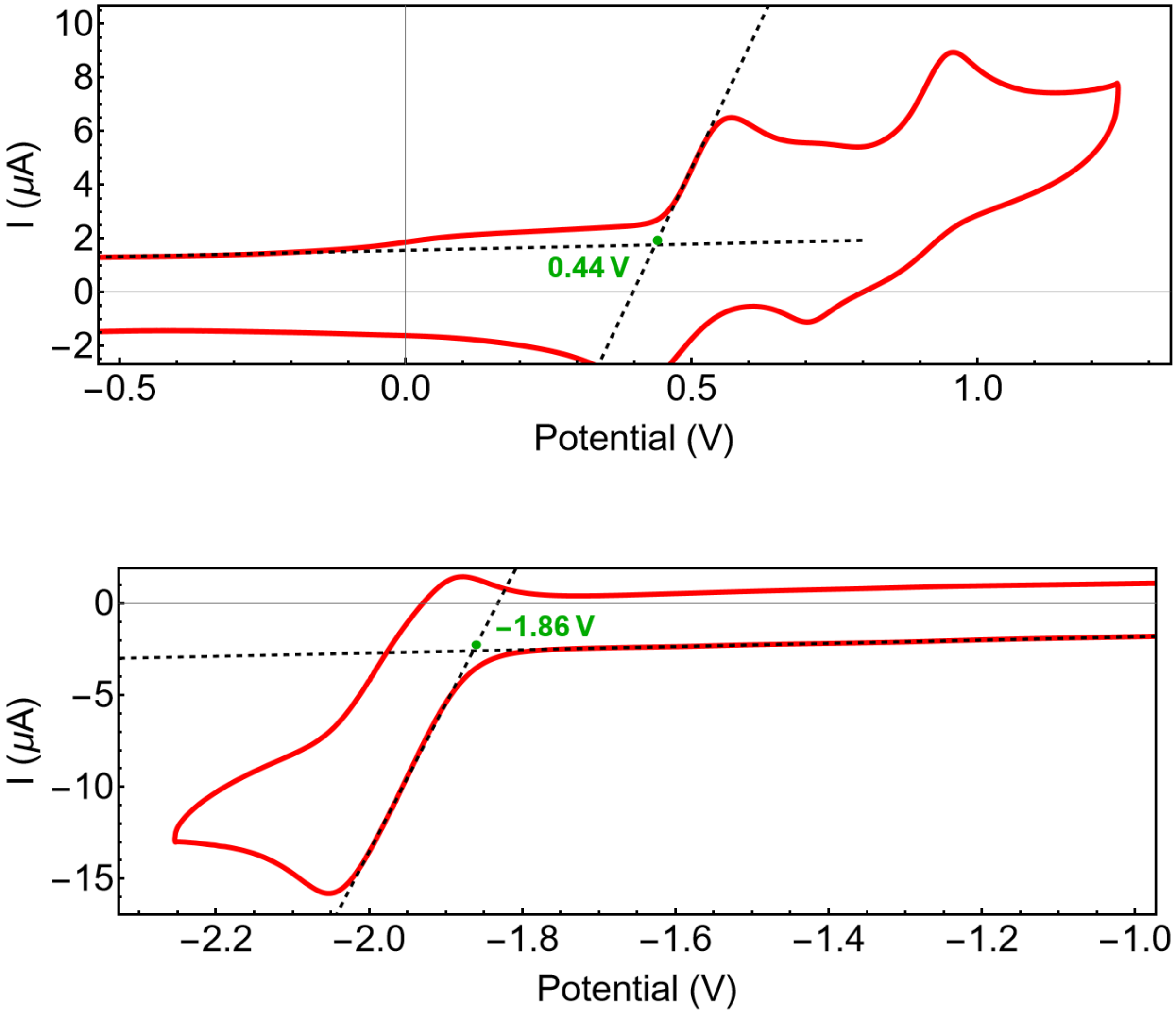

Figure S28. Determination of onset potentials for *hex*-C7FE from the CV shown in Figure S27.

Computed Ring Strain Energies

Ring strain energy for the complexes were calculated as the difference in total energies between the products and reactants ($E_{prod} - E_{react}$). All calculations were performed using the B3LYP-D3(BJ)/6-31G(d,p) level of theory with an IEFPCM solvation model in CH_2Cl_2. The total energy difference, which corresponds to the enthalpy of formation in this context, represents the ring strain energy. It is important to note that L6F, L7F and *me*-L6F and *me*-L7F have been constrained such that repeat units are all *syn*. The strain energy would change significantly from the lower energy all *anti* conformation.

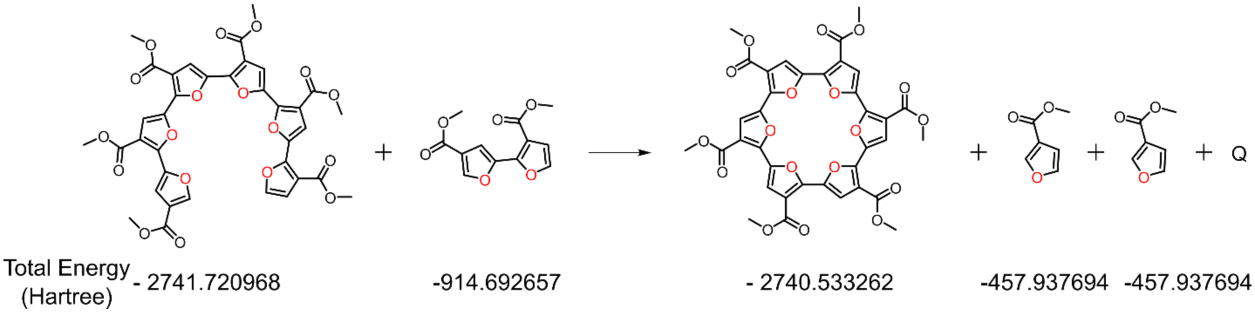

Figure S29. Ring strain energy calculation for *me*-C6FE (**3.1 kcal/mol**).

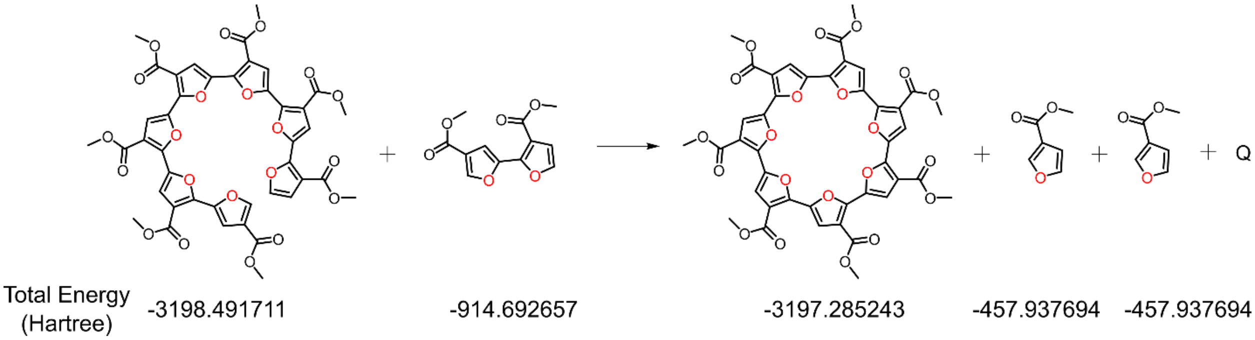

Figure S30. Ring strain energy calculation for *me*-C7FE (**14.9 kcal/mol**).

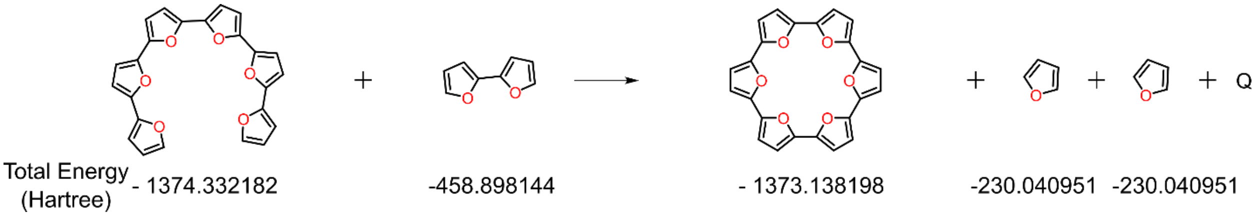

Figure S31. Ring strain energy calculation for C6F (**6.4 kcal/mol**).

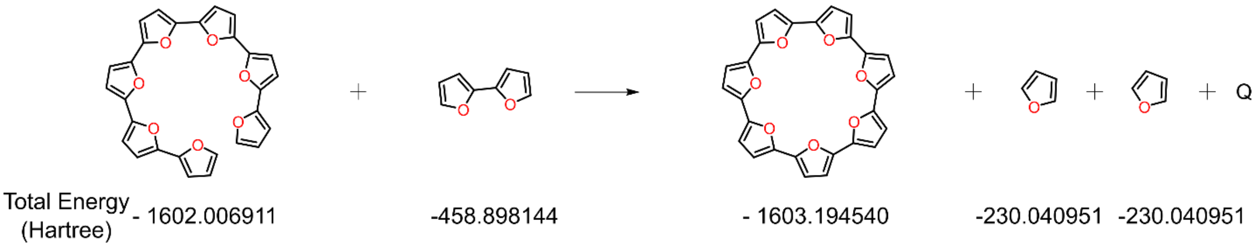

Figure S32. Ring strain energy calculation for C7F (**2.4 kcal/mol**).

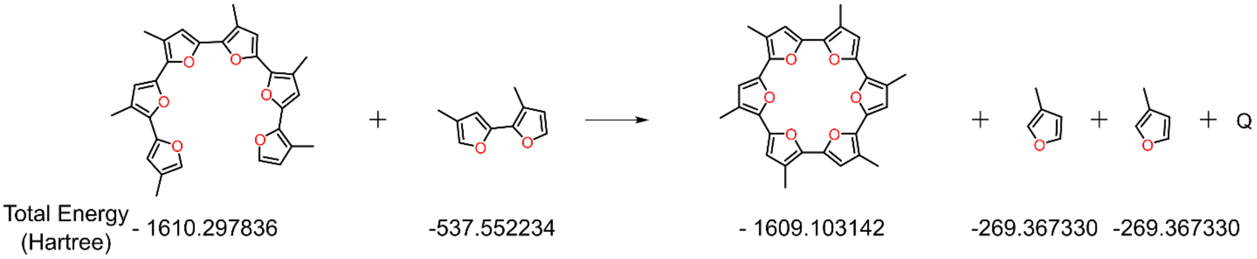

Figure S33. Ring strain energy calculation for *me*-C6F (**7.7 kcal/mol**).

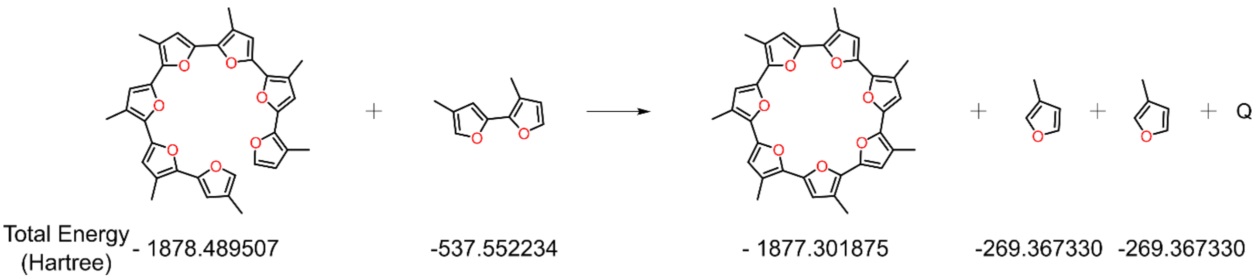

Figure S34. Ring strain energy calculation for *me*-C7F (**3.3 kcal/mol**).

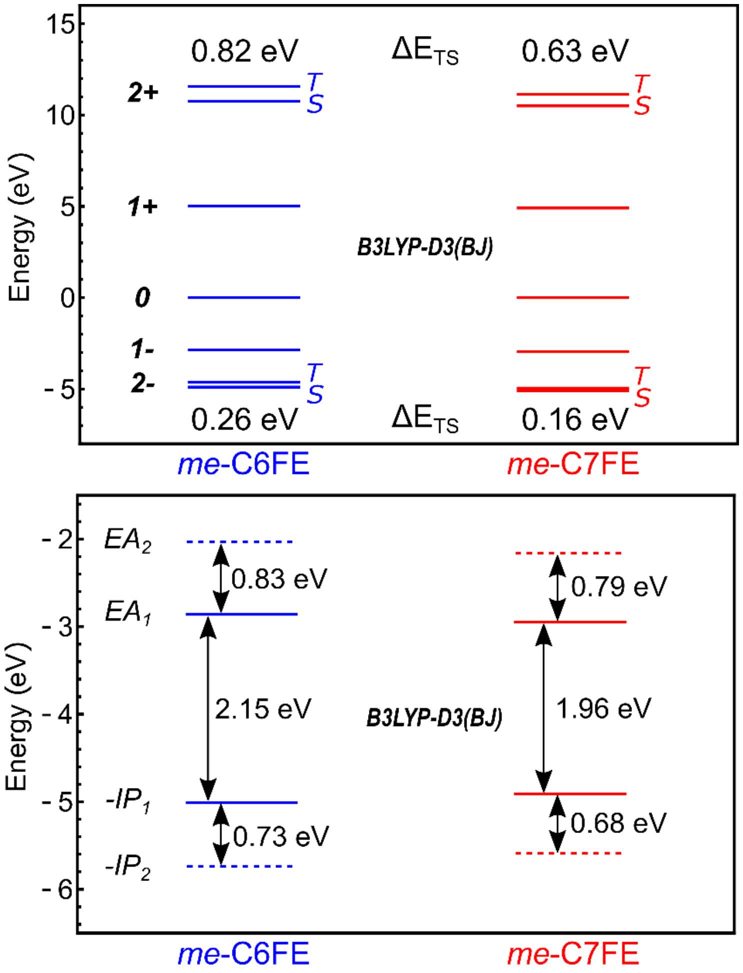

Figure S35. Total energies and frequency calculations were used to obtain Gibbs free energies (ΔG_f) for each computed structure. Top – Comparison of ΔG_f for computed redox states relative to the neutral macrocycle using B3LYP-D3(BJ)/6-31G(d,p) level of theory with a continuum solvation model (IEFPCM) in CH_2Cl_2. The computed differences between the singlet and triplet multiplicities for the +2 and −2 redox states are annotated on the plots ($\Delta E_{TS}=E_T-E_S$). Bottom – The computed differences correspond to each individual redox step where $IP_1=\Delta G_f(0)-\Delta G_f(+1)$ and $EA_1=\Delta G_f(-1)-\Delta G_f(0)$.

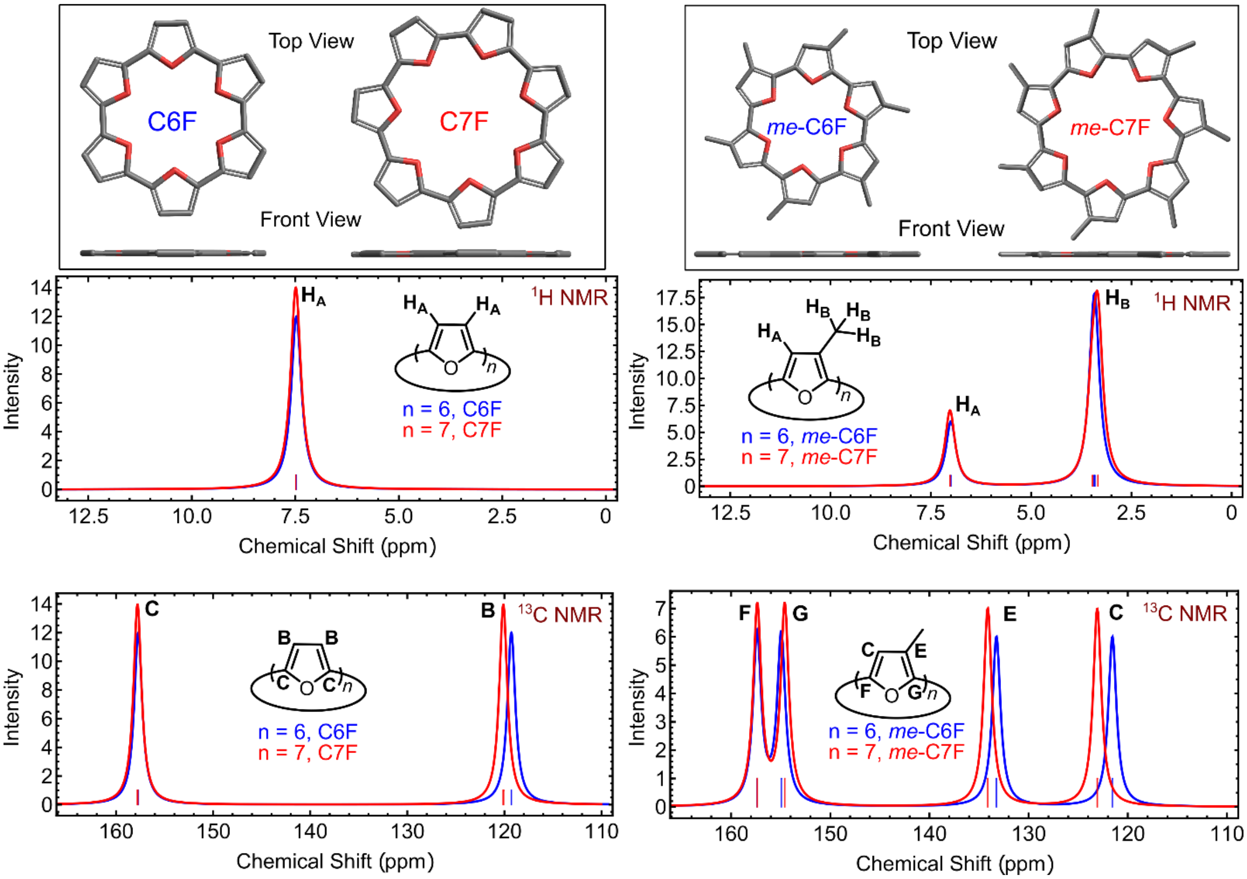

Figure S36. Optimized structures and computed ^{1}H and ^{13}C NMR chemical shifts of cyclized furan oligomers without side chains (left) and with methyl side chains (right). The structures were optimized using DFT calculations at the B3LYP-D3(BJ)/6-31G(d,p) level of theory with a continuum solvation model (IEFPCM) in CH_2Cl_2. The NMR spectra were computed using the NMR-GIAO method, employing the same B3LYP-D3(BJ) functional and 6-31G(d,p) basis set to ensure consistency between the optimized geometries and the predicted chemical shifts. Both sets of calculations used a continuum solvation model (IEFPCM) in CH_2Cl_2.

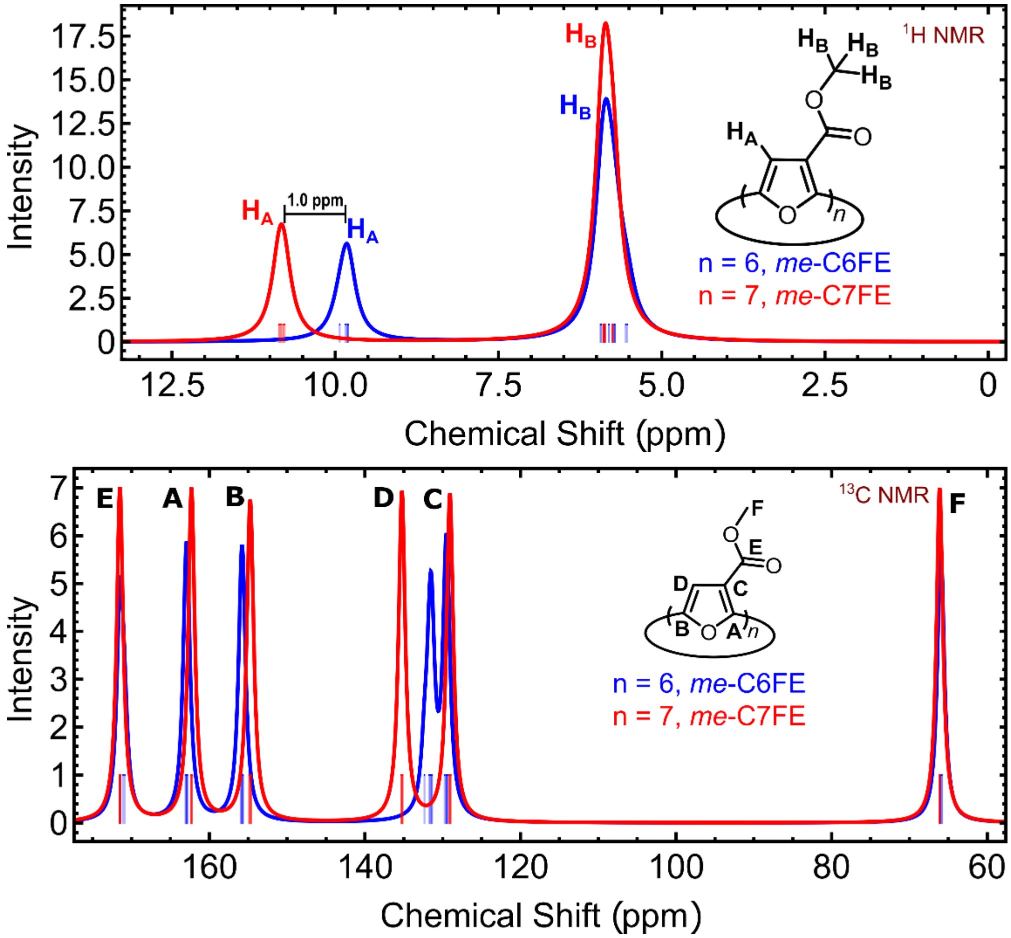

Figure S37. Computed ^{1}**H** (top) and ^{13}**C NMR** (bottom) chemical shifts of cyclized furan ester oligomers. Calculations were performed using the NMR-GIAO method with the B3LYP-D3(BJ) functional and 6-31G(d,p) basis set, employing a continuum solvation model (IEFPCM) in CH_2Cl_2.

References

(1) Varni, A. J.; Fortney, A.; Baker, M. A.; Worch, J. C.; Qiu, Y.; Yaron, D.; Bernhard, S.; Noonan, K. J. T.; Kowalewski, T. Photostable Helical Polyfurans. *J. Am. Chem. Soc.* **2019**, *141*, 8858-8867.

(2) Bruno, N. C.; Tudge, M. T.; Buchwald, S. L. Design and preparation of new palladium precatalysts for C-C and C-N cross-coupling reactions. *Chem. Sci.* **2013**, *4*, 916-920.

(3) Zalesskiy, S. S.; Ananikov, V. P. $Pd_2(dba)_3$ as a Precursor of Soluble Metal Complexes and Nanoparticles: Determination of Palladium Active Species for Catalysis and Synthesis. *Organometallics* **2012**, *31*, 2302-2309.

(4) Frisch, M. J.; Trucks, G. W.; Schlegel, H. B.; Scuseria, G. E.; Robb, M. A.; Cheeseman, J. R.; Scalmani, G.; Barone, V.; Petersson, G. A.; Nakatsuji, H.; Li, X.; Caricato, M.; Marenich, A. V.; Bloino, J.; Janesko, B. G.; Gomperts, R.; Mennucci, B.; Hratchian, H. P.; Ortiz, J. V.; Izmaylov, A. F.; Sonnenberg, J. L.; Williams; Ding, F.; Lipparini, F.; Egidi, F.; Goings, J.; Peng, B.; Petrone, A.; Henderson, T.; Ranasinghe, D.; Zakrzewski, V. G.; Gao, J.; Rega, N.; Zheng, G.; Liang, W.; Hada, M.; Ehara, M.; Toyota, K.; Fukuda, R.; Hasegawa, J.; Ishida, M.; Nakajima, T.; Honda, Y.; Kitao, O.; Nakai, H.; Vreven, T.; Throssell, K.; Montgomery Jr., J. A.; Peralta, J. E.; Ogliaro, F.; Bearpark, M. J.; Heyd, J. J.; Brothers, E. N.; Kudin, K. N.; Staroverov, V. N.; Keith, T. A.; Kobayashi, R.; Normand, J.; Raghavachari, K.; Rendell, A. P.; Burant, J. C.; Iyengar, S. S.; Tomasi, J.; Cossi, M.; Millam, J. M.; Klene, M.; Adamo, C.; Cammi, R.; Ochterski, J. W.; Martin, R. L.; Morokuma, K.; Farkas, O.; Foresman, J. B.; Fox, D. J. *Gaussian 16 Rev. C.01*; Wallingford, CT, 2016. https://gaussian.com/.

(5) Glendening, E. D.; Landis, C. R.; Weinhold, F. NBO 7.0: New vistas in localized and delocalized chemical bonding theory. *J. Comput. Chem.* **2019**, *40*, 2234-2241.

(6) Lu, T.; Chen, F. W. Multiwfn: A multifunctional wavefunction analyzer. *J. Comput. Chem.* **2012**, *33*, 580-592.

(7) Chen, Z.; Wannere, C. S.; Corminboeuf, C.; Puchta, R.; Schleyer, P. R. Nucleus-Independent Chemical Shifts (NICS) as an Aromaticity Criterion. *Chem. Rev.* **2005**, *105*, 3842-3888.
